# dJun and Vri/dNFIL3 Are Major Regulators of Cardiac Aging in Drosophila

**DOI:** 10.1371/journal.pgen.1003081

**Published:** 2012-11-29

**Authors:** Véronique Monnier, Magali Iché-Torres, Michael Rera, Vincent Contremoulins, Céline Guichard, Nathalie Lalevée, Hervé Tricoire, Laurent Perrin

**Affiliations:** 1 Unité de Biologie Fonctionnelle et Adaptative (BFA) EAC4413 CNRS, Université Paris Diderot, Sorbonne Paris Cité, Paris, France; 2 Technologies Avancées pour le Génome et la Clinique (TAGC), UMR 1090 INSERM- Université d'Aix-Marseille, Parc Scientifique de Luminy, Case 928, Marseille, France; 3 IBDML, UMR6216 CNRS-Université d'Aix-Marseille, Parc Scientifique de Luminy, Case 907, Marseille, France; 4 ImagoSeine, Institut Jacques Monod, UMR 7592, CNRS and Université Paris-Diderot, Paris, France; Stanford University Medical Center, United States of America

## Abstract

Cardiac aging is a complex process, which is influenced by both environmental and genetic factors. Deciphering the mechanisms involved in heart senescence therefore requires identifying the molecular pathways that are affected by age in controlled environmental and genetic conditions. We describe a functional genomic investigation of the genetic control of cardiac senescence in Drosophila. Molecular signatures of heart aging were identified by differential transcriptome analysis followed by a detailed bio-informatic analysis. This approach implicated the JNK/dJun pathway and the transcription factor Vri/dNFIL3 in the transcription regulatory network involved in cardiac senescence and suggested the possible involvement of oxidative stress (OS) in the aging process. To validate these predictions, we developed a new *in vivo* assay to analyze heart performance in various contexts of adult heart-specific gene overexpression and inactivation. We demonstrate that, as in mammals, OS plays a central role in cardiac senescence, and we show that pharmacological interventions impinging on OS slow heart senescence. These observations strengthen the idea that cardiac aging is controlled by evolutionarily conserved mechanisms, further validating Drosophila as a model to study cardiac senescence. In addition, we demonstrate that Vri, the ortholog of the vertebrate NFIL3/E4B4 transcription factor, is a major genetic regulator of cardiac aging. Vri overexpression leads to major heart dysfunctions, but its loss of function significantly reduces age-related cardiac dysfunctions. Furthermore, we unambiguously show that the JNK/AP1 pathway, the role of which in cardiac aging in mammals is controversial, is activated during cardiac aging and has a detrimental effect on cardiac senescence. This data-driven functional genomic analysis therefore led to the identification of key components of the Gene Regulatory Network of cardiac aging in Drosophila and may prompt to investigate the involvement of their counterparts in the cardiac aging process in mammals.

## Author Summary

Age-associated changes in cardiac structure and function have been implicated in the markedly increased risk of cardiovascular disease, but the molecular basis of these processes is ill-defined. It is difficult to study the genetics of heart aging in mammalian models because of their long life spans and their complexity, involving notably genetic redundancy. Here, we address this issue through identification of molecular signatures of cardiac aging in Drosophila, a model organism in which heart senescence occurs within 2 months. Tissue-specific transcriptome comparison of young and aging fly hearts were performed followed by *in silico* predictions of the regulatory networks involved. This analysis implicated oxidative stress (OS), the JNK/dJun pathway, and Vri/dNFIL3 in the gene regulatory network that drives cardiac senescence. Measuring heart variables *in vivo* following heart-specific genetic and pharmacological manipulations confirmed these predictions. We show that OS has a central role in the aging of the fly heart. Moreover, heart-specific partial knockdown of dJun and Vri prevented cardiac senescence, demonstrating that they are essential regulators of cardiac aging. Thus, our results uncover two major genetic determinants of Drosophila cardiac aging whose activities enhance heart senescence. It may therefore be valuable to investigate their involvement in the cardiac aging process in mammals.

## Introduction

Age-associated changes in cardiac structure and function may contribute to the markedly increased risk for cardiovascular disease, what urges the need to understand heart aging at the molecular level. Cardiac senescence is a complex process, characterized by impaired cardio-acceleration during stress (cardiac reserve) and increased arrhythmia; it involves interactions between age, lifestyle, various diseases and genetic components [1]. Addressing the genetic basis of heart aging in mammalian models is challenging, due to their long life span, the complexity of the process (many genes may be involved), the complexity of the models themselves (genetic redundancy) and the complex interactions between genetic traits and age/disease/lifestyle. There is therefore a need for simpler model organisms where genetic components of cardiac senescence can be more readily and rapidly assessed.

Drosophila has recently emerged as an attractive model of cardiac aging [2]. Drosophila develops to adulthood quickly, has a relatively short life span (50 to 70 days at 25°C) and is a highly tractable genetic system. The feasibility of analyzing large cohorts of individuals in tightly controlled environments makes it a powerful model for aging studies. Analogous to observations in elderly humans, the maximal heart rate is significantly lower in old than young Drosophila [3], and an age-associated increase in rhythm disturbances has been observed. Wessells *et al* [4] demonstrated that modulating the insulin signaling pathway (which has a conserved role in regulating life span [5]) exclusively in the fly heart prevents the decline in cardiac performance with age. Several recent reports further support the use of Drosophila for analyzing cardiac responses to age-related stress (reviewed in [2]). For example, the K^+^_ATP_ channel encoded by the *dSur* gene appears to play a protective role against hypoxic stress, very much like its vertebrate counterpart, and *dSur* expression decreases with age, consistent with it being involved in the heart aging phenotype [6]. However, in spite of these pioneering studies, the molecular pathways involved in the progressive modifications of heart performance with age, and their genetic and environmental control, are still poorly defined.

Here, we describe a functional genomic approach to investigate the genetic control of cardiac senescence in Drosophila. First, we identified molecular signatures of heart aging by differential transcriptome analysis of young (10 days) and aging (40 days) adult hearts. Data mining, which included prediction of transcription regulatory networks [7], suggested that the JNK/AP1 pathway and the Vri/NFIL3 transcription factors are central to regulating cardiac senescence. This analysis also identified a potential role of oxidative stress (OS). These predictions were tested by analyzing measures of heart senescence *in vivo* in flies following heart-specific genetic manipulations. In particular, the JNK/AP1 pathway was clearly found to be activated during cardiac aging and to have a detrimental effect on cardiac senescence. Furthermore, we demonstrate that the transcription factor Vri/dNFIL3 is a major genetic regulator of cardiac aging. Vri overexpression led to major heart dysfunction and its loss of function significantly reduced cardiac senescence. Thus, our study reveals two major genetic determinants of Drosophila cardiac aging, and paves the way for further studies in mammals.

## Results/Discussion

### Identification of cardiac aging signatures by differential transcriptome analysis

To identify molecular signatures of cardiac aging, we performed a differential transcriptome analysis to compare young (10 days) and aging (40 days) hearts. At age 40 days, manifestations of cardiac senescence are clearly visible [3], [4], although flies still exhibit low mortality (<10%) in all genetic conditions tested. Flies were raised in tightly controlled conditions and RNA extracted from dissected hearts was used to probe microarrays (see experimental procedures for details): 3097 probes representing 1107 unique Drosophila genes were found to be differentially expressed between the two time points, including 635 genes induced and 472 repressed at age 40 days (Table 1 and Table S1 and S2, full transcriptome data are accessible in the Gene Expression Omnibus database under the accession number GSE40253).

**Table 1:** Molecular pathways and putative transcription regulatory networks affected during cardiac aging.

	N° of genes(1)	GO term enrichment(2)	Oxidative Stress		JNK pathway(5)	CisTargetX Analysis(6)
			MnSOD Transcriptome Enrichment(3)	Paraquat Transcriptome Enrichment(4)		
**Induced**	635	defense response	147/1025	63/538	29/133	AP1/Jun (z = 6.78)
**(cluster 2)**		response to stress	p<10^−20^	p<2 10^−5^	p<10^−7^	Evi1 (z = 6.08)
		vesicle-mediated transport				GATA (z = 4.08)
		carbohydrate catabolic process				
**Repressed**	472	cellular respiration	32/1305	37/611	nd	Vri/NFIL3 (z = 6.12)
**(cluster 1)**		electron transport chain	P = 0.3	P = 0.15		
		hexose metabolic process				

Overview of differential gene expression analysis between 10 days and 40 days adult cardiac tubes. Enrichment p values were calculated using a test based on hypergeometric distribution.

(1) Statistically significantly deregulated genes between 10 and 40 day-old dissected hearts were determined using Limma software from normalized expression data (See Table S1 and S2).

(2) GO term enrichments were determined using the Flymine database (www.flymine.org) (see Table S5 for details).

(3) [13]; See Table S6 for details.

(4) [14]; See Table S7 for details.

(5) [17], [18]; see Table S9 for details.

(6) Transcriptional regulatory network were predicted with cisTargetX (http://med.kuleuven.be/cme-mg/lng/cisTargetX/.) The z score of the most representative TF-binding motif is indicated for each motif family. (See Table S8 for details).

To validate the microarray expression data by quantitative real-time PCR, seven genes with different expression profiles (3 down regulated and 4 up regulated) were selected. In all cases tested, the changes observed in the arrays were confirmed (Figure S1).

Almost half of the genes identified as being differentially expressed (523, 47%) have previously been described as age responsive in previous studies focused on whole individual, or other body parts (Table S3); these genes therefore contribute to molecular signatures common to aging. However, more than half of the genes that were differentially expressed with age in the heart had not previously been identified as being age related. This indicates that the aging of the heart, and probably other organs, may present particular traits and confirms the relevance of tissue-specific transcriptome analysis.

Differentially expressed genes were analyzed for Gene Ontology (GO) term enrichment (Table 1 and Table S4). The set of under-expressed genes (cluster 1) was enriched for genes involved in cellular respiration and mitochondrial bioenergetics (Table S5), including those encoding mitochondrial proteins involved in electron transport. This appears to be a general signature of aging tissues in fly, mouse and humans [8]. The set of genes induced during cardiac aging (cluster 2) was enriched for inflammation and immune defense genes, another general trend of aging tissue in flies [9], [10] and mice [11]. Genes involved in carbohydrate metabolism also show significant changes during aging, indicating a putative modification of energy metabolism.

Increased oxidative stress (OS) has repeatedly been linked to the aging process (see [12] for recent review) and the down-regulation of mitochondria-related processes observed here (Table S2 and S5) may signal impaired mitochondrial function in the aging heart. We therefore compared heart-deregulated genes with published transcriptome analyses of oxidative stress-responsive genes in the adult fly [13] [14]. One-hundred-and-forty up-regulated genes in the aged heart were found to be also up-regulated in mild MnSOD overexpression conditions ([13], enrichment, p<10^−20^) that induce a mild H_2_O_2_ -mediated OS (Table 1 and Table S6). In addition, 63 up-regulated genes were also found to be activated following oxidative stress induced by paraquat treatment ([14]; enrichment p<5 10^−5^, Table S7). These observations suggest that oxidative stress plays an important role in Drosophila heart aging. This notion was further supported by cardiac-specific manipulation of Catalase expression, a reactive oxygen species-scavenging enzyme (see below).

### Regulatory network predictions suggest that cardiac aging is regulated by AP1 and Vri/dNFIL3

Genes differentially expressed with age may be regulated by transcription factors (TFs) which would constitute good candidates for the control of heart senescence. To investigate the transcriptional regulation of age-regulated genes, we performed *in silico* predictions of TF-binding motifs potentially involved in the regulation of genes up- and down-regulated in the aging heart. We used the recently published *cis*TargetX method [7], [15]. *cis*TargetX uses identification of clusters of TF-binding motifs across the entire genomes of 12 Drosophila species and ranking statistics, to determine significant associations between motifs and subsets of co-expressed genes. It uses a large sequence space (5 kb up to the 5′ transcription start, and the introns of all genes) and a comprehensive library of TF-binding motifs to predict potential regulatory motifs. We reasoned that it might therefore allow regulatory motif predictions without prior knowledge of the TFs involved. Motifs known to bind AP1 –the Fos/Jun heterodimer— were substantially enriched in the subset of over-expressed genes (z-score = 6.30) defining a set of 98 potential AP1 targets (Table 1 and Table S8). Interestingly, AP1 is an established effector of the Jun N-terminal Kinase (JNK) signaling pathway, which is one of the most versatile stress sensors in metazoans and has been linked to aging in fly [16]. The JNK pathway in Drosophila involves Basket (Bsk, dJNK) and an additional kinase, Hemipterous, which serves to phosphorylate Bsk. Consistent with a role for the JNK pathway in cardiac aging, 22% (29/133, p<10^−7^) of JNK targets previously identified in Drosophila S2 cells following either bsk or Hep inactivation [17], [18] are present among the genes up-regulated at 40 days (Table 1 and Table S9). Interestingly, Jra, the Jun Drosophila orthologue, is one of these genes, suggesting a positive feedback loop of AP1 activation in the aging heart. These various findings strongly suggest that the JNK-AP1 pathway is involved in Drosophila heart aging.

*cis*TargetX also identified Evi-1 motifs as being potentially involved in the regulation of over-expressed genes during Drosophila heart aging. Evi-1 motifs –characterized as binding the vertebrate zinc finger TF Evi-1— were greatly enriched (z = 6.1) in the conserved non-coding sequences of this gene set (Table 1 and Table S8). A list of 30 genes potentially regulated by these motifs during heart aging is given in Table S8. However, the corresponding drosophila TF has not been unambiguously identified: the drosophila genome contains two genes encoding Evi-1 homologues –*hamlet* (*ham*), which regulates cell identity in the peripheral nervous system [19] and *CG10348* of unknown function- and their binding motifs have not been characterized. In addition, Evi-1 motifs are constituted of tandem repeats of GATA motifs, such that their significant association with up-regulated genes may alternatively indicate the involvement of TFs of the GATA family.

In contrast to the set of age-induced genes, we did not identify enrichment in AP1 or Evi-1 motifs in the set of down-regulated genes. However we found a strong enrichment (z = 6.5) for motifs associated with the bZip TF Vrille (Vri) and its mammalian homologue NFIL3 (also called E4BP4), which act mainly as a transcriptional repressors [20] (Table 1 and Table S8). Both Vri and NFIL3 have been implicated in many developmental processes but Vri/NFIL3 has never previously been reported to be associated with aging either in vertebrates or in Drosophila.

These *in silico* predictions of TF-binding motifs thus suggest that Vri, dJun and an un-identified TF able to bind Evi-1 motifs participate in the transcriptional regulatory network involved in heart senescence.

### A new *in vivo* assay to analyze cardiac senescence in Drosophila

**Figure 1:**
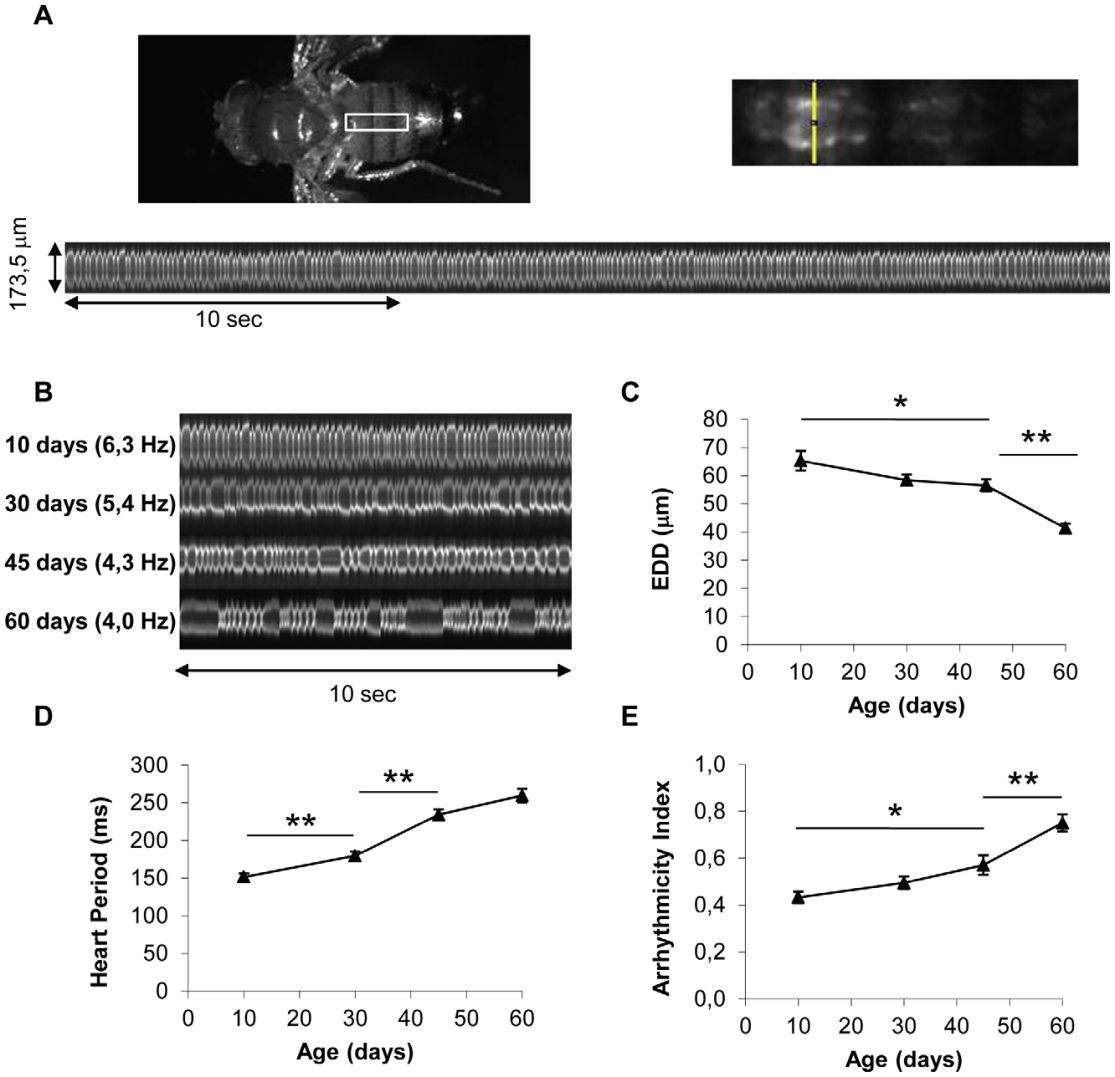
*In vivo* analysis of cardiac aging. (A) Flies expressing a GFP protein targeted to mitochondria under control of the Heart-specific GeneSwitch protein (*w/Y;UAS-mitoGFP/+; Hand-GS/+* male flies treated with RU486) were anaesthetized and fixed by their wings. Videos were acquired under a Stereomicroscope (1000 frames per movie, 32 frames/s). M-Mode was generated by horizontal alignment of rows extracted at the same position from each movie frame, with automated positioning of the acquisition zone (yellow segment). (B) Representative 10 second M-Modes of hearts at various ages. Cardiac frequency is indicated. (C,D,E) End Diastolic Diameter (EDD, µm), Heart Period (ms) and Arrhythmicity Index of 10-day-old (n = 26), 30-day-old (n = 34), 45-day-old (n = 34) and 60-day-old (n = 32) flies. All values are means (±SEM). Significant differences between successive ages are indicated: * p<5.10^−2^, ** p<5.10^−3^.

We developed a new *in vivo* assay to analyze heart performance in various contexts of adult heart-specific gene overexpression and inactivation. Our aim was to set up an assay suitable for large-scale analysis, allowing conditional and tissue-specific genetic manipulations and measurements of heart senescence in physiological conditions in intact (not dissected) individuals. We exploited the GeneSwitch system [21] which allows expression to be induced by RU486 feeding. A heart-specific Geneswitch driver, Hand-GS, was constructed and combined with a UAS-mitoGFP construct. Hand-GS>UAS-mitoGFP flies fed with RU486 exhibit a cardiac fluorescence strong enough for high-speed video recording through the cuticle of anaesthetized flies of various ages (Figure 1A). Flies imaged under UV light exhibit a heart rate (HR) in the same range as those obtained in previous studies on intact flies [22], [23], [24]. HR under UV is slightly higher and more regular than the mean HR obtained under visible light on anesthetized flies (Figure S2). Image acquisition and analysis of M-Modes are described in details in Text S1 and Figure S2. Relevant functional measures were extracted from M-Mode analysis and used to quantify heart performance, including the mean time between successive end-diastolic positions (Heart Period, HP), an Arrhythmicity Index (AI) defined as the standard deviation of the HP normalized to the median HP as described in [25] and the End-Diastolic Diameter (EDD). We checked that heart performance was not dependent on RU concentration at any age (comparison of two RU486 treatments −20 µg/ml and 100 µg/ml- was performed), and was not affected by expression of luciferase or a control ds-RNA (Figure S3).

We observed a progressive increase in HP with age, mainly between age 10 days and 45 days, and an increased AI mainly between ages 45 days and 60 days (Figure 1B, 1D and 1E, Video S1, S2 and S3). Similar age-related decreases in heart performance have been described in flies using other methods of heart beat detection, including semi-intact preparations, in which the heart is surgically exposed [3], [4], [25]. In addition, we observed a progressive decrease in End-Systolic Diameter (Data not shown) and End-Diastolic Diameter (EDD, Figure 1C). EDD decreased by 13,4% between ages 10 and 45 days, and by 26,9% between ages 45 and 60 days.

Our assay appears to have various advantages over previously used methods for studying cardiac aging. First, heart activity is monitored in intact flies, in optimized physiological conditions. Second, it does not require highly specialized equipment and is suitable for large-scale analysis since movie acquisition is fast (30 flies can be recorded in one hour) and each step of the analysis is automated. Finally, the heart-specific inducible driver provides the opportunity to study adult-specific gene overexpression or ds-RNA mediated inactivation, independently of other developmental effects.

### Genetic and pharmacological modulation of OS can prevent cardiac senescence

**Figure 2:**
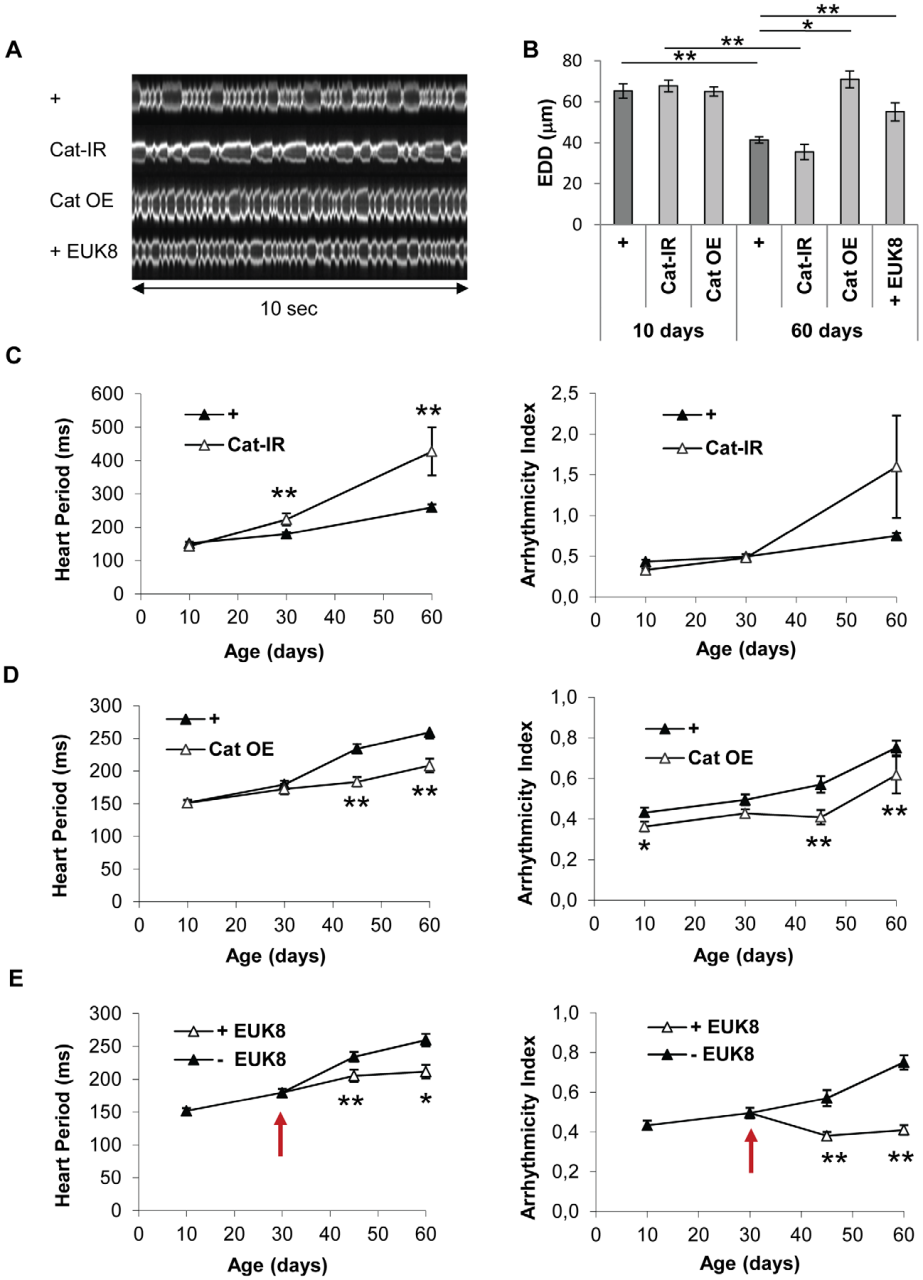
Catalase overexpression and treatment of wild-type flies with a catalase/SOD mimetic improve heart performance. Cardiac imaging was performed on *w/Y;UAS-mitoGFP/+; Hand-GS/+* (+), *w/Y;UAS-mitoGFP/+; Hand-GS/UAS-Catalase-IR* (Cat-IR), *w/Y;UAS-mitoGFP/UAS-Catalase; Hand-GS/+* (Cat OE) male flies. *w/Y;UAS-mitoGFP/+; Hand-GS/+* male flies were treated with EUK-8 (0.2 mM) from the age of 30 days (+ EUK-8, red arrow shows the start of the treatment). All flies were treated with RU 486 (100 µg/ml of food) during adulthood. (A) Representative 10 second M-Modes of 60-day-old flies. (B) End Diastolic Diameter (EDD, µm) in 10-day-old and 60-day-old flies. Significant differences are indicated: * p<5.10^−2^, ** p<5.10^−3^. (C,D,E) Heart Period (ms) and Arrhythmicity Index. Significant differences with control (+) flies of the same age are indicated: * p<5.10^−2^, ** p<5.10^−3^. All values are means (±SEM). +/10 days: n = 26; +/30 days: n = 34; +/45 days: n = 34; +/60 days: n = 32; Cat-IR/10 days: n = 10; Cat-IR/30 days: n = 10; Cat-IR/60 days: n = 10; Cat OE/10 days: n = 19; Cat OE/30 days: n = 9; Cat OE/45 days: n = 19; Cat OE/60 days: n = 17; +EUK-8/45 days: n = 19; +EUK-8/60 days: n = 7.

The transcriptome analyses suggested that the fly heart is subject to rising levels of oxidative stress with age (see above). This was confirmed by manipulating Catalase activity, an antioxidant enzyme which detoxify H2O2, specifically in the cardiac tube at adulthood, and by analyzing the induced transcriptome modifications at 40 days (Table 2 and Table S12). Genes whose expression is induced following increased OS –eg induced in RNAi mediated Cat knockdown compared to Cat overexpression- are highly enriched in genes that are overexpressed in cardiac tubes at 40 days compared to 10 days (cluster 2; 59/635 p = 4.9 10^−35^) and is enriched in genes participating in defense response (Table S13). On the contrary, the set of genes that are up regulated following reduced OS -eg in Cat overexpressing hearts compared to Cat knockdown- is highly significantly enriched in cluster 1 genes (gene set downregulated at 40 days compared to 10 days, 119/472, p = 3.22 10^−68^). This analysis thus confirms the central role played by OS in the age related cardiac transcriptome dynamics. We therefore directly tested the involvement of oxidative stress in cardiac aging by genetically modulating the expression of the antioxidant enzymes Superoxide dismutase (SOD) which detoxify O2•– and Catalase in the heart and analyzed heart parameters. Catalase inactivation (Cat-IR condition) led to a strong age-dependent deleterious phenotype. HP was 71% higher in 60-day-old flies than controls of the same age (Figure 2A and 2C, Video S4, Table S11). Inversely, over-expression of Catalase (Cat OE condition) substantially improved cardiac performance in old flies (Figure 2A and 2D, Video S5), with lower HP and arrhythmia in both 45 and 60 days old flies compared to controls. Expression of another enzyme with catalase activity (Catalase B, CG9314), normally not expressed in cardiac tissues, also improved cardiac function (Figure S4A), confirming the beneficial effect of catalase activity for preventing cardiac senescence. There were no significant differences in heart performance, at any individual time-points, between flies with enhanced or decreased expression of SOD1 (CuZn or cytosolic SOD) (Figure S4B and S4C), although the rate of AI increase as function of age was slightly higher in SOD1 depleted flies (Table S11). Altogether, this suggests that H2O2-mediated oxidative stress is predominant in the cardiac aging process. Next, we treated wild type flies with EUK-8 (+ EUK8 condition), a synthetic superoxide dismutase and catalase mimetic, from the age of 30 days, and compared heart performance between treated and untreated 45 and 60 day-old flies. EUK-8 improved both HP and AI (Figure 2A, 2E, Video S6 and Table S11). In particular, it fully abolished the age-dependent development of cardiac arrhythmia: the AI was not different in 10-day-old and EUK-8-treated 60 day-old flies (p = 0.3), whereas AI increased by 74% in control flies between ages 10 and 60 days (p<5.10^−3^). EUK-8 treatment also prevented the age-related EDD decrease (Figure 2B): the EDD was not different (p = 0,26) between 30 days (start of treatment) and 60 days old treated flies, whereas EDD decreased by 29% (p<5.10^−3^) between these two ages in untreated flies. Similarly, flies overexpressing Catalase presented a stable EDD between ages 10 days and 60 days (p = 0,13).

**Table 2:** Effect of Catalase activity on cardiac transcriptome at 40 days.

	N° of genes(1)	GO term enrichment(2)	Cluster 1 Enrichment(3)	Cluster 2 Enrichment(4)	JNK pathway enrichment(5)
**Induced in Cat LOF versus GOF**	187	Defense response	4/472p = 0.76	59/635p = 4.9 10^−35^	1/133p = 0.53
**Repressed in Cat LOF versus GOF**	521	Proteolysis	119/472p = 3.22 10^−68^	20/635p = 0.73	3/133p = 0.73

Overview of genes deregulated in 40 days old cardiac tubes between Catalase Loss of function (LOF) and Gain of Function (GOF). Enrichment p values were calculated using a test based on hypergeometric distribution.

(1) Deregulated genes between Hand-GS/UAS>Ca-IR (Cat LOF) and Hand-GS/UAS>Catalase (Cat GOF) 40 days-old cardiac tubes dissected from flies fed with 100 µg/ml RU486 were determined using Rank Product software from normalized expression data (see Table S12).

(2) GO term enrichments were determined using the Flymine database (www.flymine.org) (see Table S13 for details).

(3) See Table 1 and Table S2 (repressed genes at 40 days compared to 10 days).

(4) See Table 1 and table S2 (induced genes at 40 days compared to 10 days).

(5) [17], [18].

Interestingly, in mammals, expression of mCAT, a catalase artificially targeted to mitochondria, protects mice from cardiac aging [26]. EUK-8 or other synthetic SOD/Cat mimetic also appear to improve heart function in pathological contexts [27] [28] [29]. Our findings and these various observations highlight the role of oxidative stress in both pathological and normal cardiac aging and indicate that mechanisms of cardiac aging are conserved between flies and mammals. In addition, we show here that pharmacological antioxidant strategies can improve heart function in old individuals.

### dJun and Vri/dNFIL3 are major regulators of heart senescence

**Figure 3:**
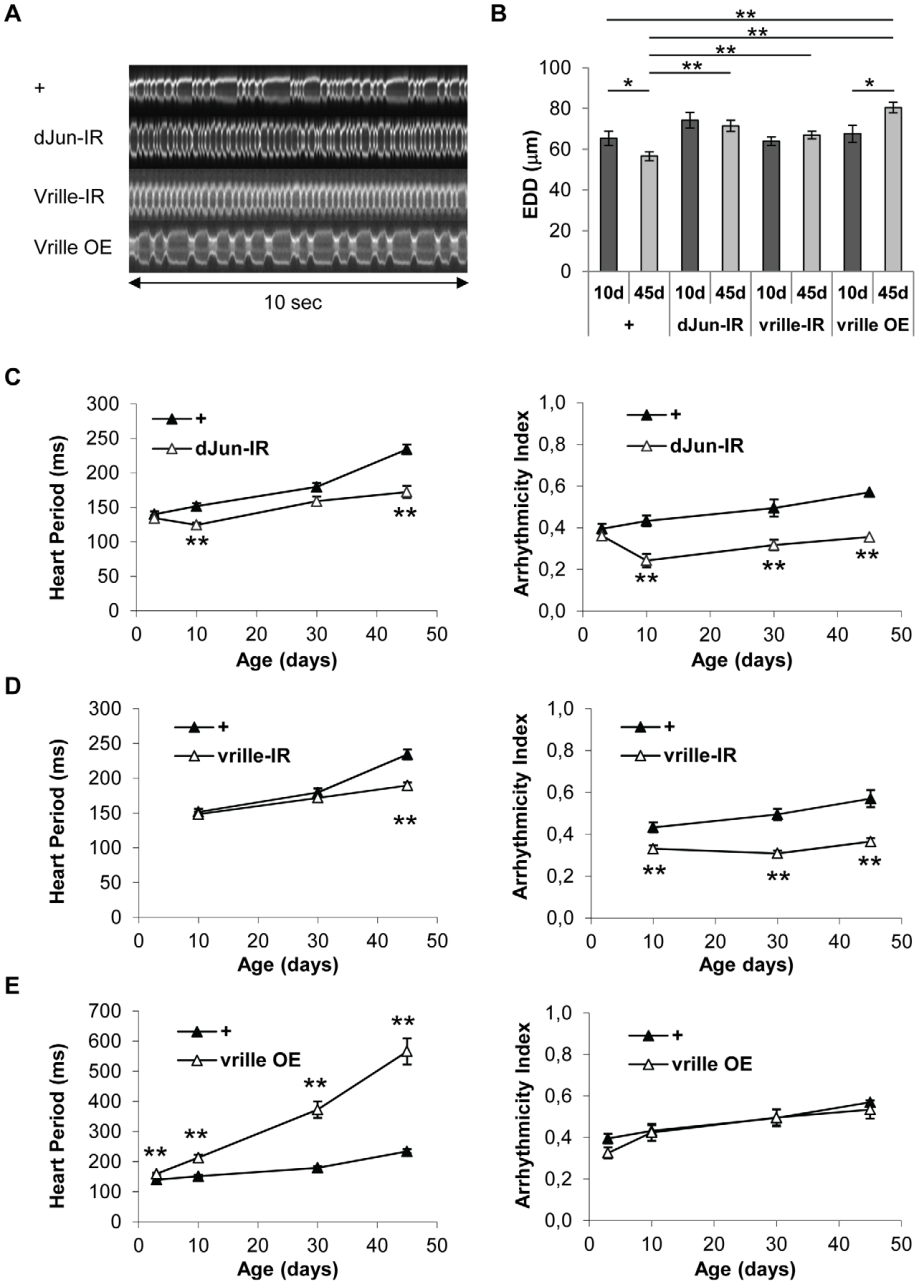
Heart-specific inactivation of dJun and Vrille transcription factors improve heart performance. Cardiac imaging was performed on *w/Y;UAS-mitoGFP/+;Hand-GS/+* (+), *w/Y;UAS-mitoGFP/+;Hand-GS/UAS-dJun-IR* (dJun-IR), *w/Y;UAS-mitoGFP/UAS-vrille_IR; Hand-GS/+* (vrille-IR) and *w/Y;UAS-mitoGFP/UAS-vrille; Hand-GS/+* (vrille OE) male flies treated with RU 486 (100 µg/ml of food) during adulthood. (A) Representative 10 sec M-Modes of 45-day-old flies. (B) End Diastolic Diameter (EDD, µm) in 10-day-old and 45-day-old flies. Significant differences are indicated: * p<5.10^−2^, ** p<5.10^−3^. (C,D,E) Heart Period (ms) and Arrhythmicity Index. Significant differences with control (+) flies of the same age are indicated: * p<5.10^−2^, ** p<5.10^−3^. All values are means (±SEM). +/3 days: n = 13; +/10 days: n = 26; +/30 days: n = 34; +/45 days: n = 34; dJun-IR/3 days: n = 11; dJun-IR/10 days: n = 10; dJun-IR/30 days: n = 8; dJun-IR/45 days: n = 14; vrille-IR/10 days: n = 22; vrille-IR/30 days: n = 29; vrille-IR/45 days: n = 38; vrille OE/10 days: n = 12; vrille OE/10 days: n = 3; + vrille OE/30 days: n = 15; vrille OE/45 days: n = 12.

The heart-specific inducible driver can be used to study adult-specific gene over-expression or ds-RNA-mediated inactivation, independently of other developmental effects. This allowed us to test the *in silico* predictions of a transcriptional regulatory network potentially involving dJun and Vri as regulators of cardiac senescence. A large number of JNK-responsive and potential AP1 target genes, as well as *dJun* itself, were up-regulated in the aging fly heart, suggesting that the JNK/AP1 pathway is involved in their transcriptional up regulation (cluster 2, see above). To confirm the functional involvement of *dJun* in the transcriptional deregulation of these up-regulated genes, we performed a transcriptome analysis of 40 days old hearts following *dJun* knockdown by dsRNA and focused on genes whose expression is repressed in *dJun* knockdown compared to control cardiac tubes (Table 3 and Table S14). Not surprisingly, the set of genes whose expression level is reduced by at least 1.5 fold following *dJun* knockdown is significantly enriched in JNK target genes (29/135 genes, p = 1.1 10^−5^). Importantly, this gene set was also highly significantly enriched in cluster 2 genes (138/635 genes, p = 10^−19^), what support a function for *dJun* in the age-related transcriptome deregulation reported above. In addition, the predicted AP1 targets within cluster 2 (Table S8) were also enriched in this gene set: almost 25% of predicted *cis*targetX AP1 targets are under-expressed after *dJun* knockdown (23/99, p = 1.8 10 ^−5^). Altogether, these data validate the *cis*TargetX predictions made from cluster 2 and confirm the central role played by the JNK/AP1 pathway in the up regulation of a significant proportion of genes in aging hearts. We therefore used ds-RNA-mediated inactivation to abolish *dJun* activity in the heart (dJun-IR condition) and measured heart parameters. This significantly improved heart function and led to a higher heart rate and less arrhythmia (Figure 3A and 3C, Video S7), in both young (10-day-old) and aged (45-day-old) flies than in the corresponding controls. HP and AI were respectively 26.5% and 37.6% lower in 45-day-old flies than in controls of the same age. Noticeably, HP and AI were similar in 3 days old dJun-IR and control flies. In addition to the beneficial effects already observed in 10 days old flies, *dJun* partial inactivation significantly slowed down the rate of HP increase as a function of age (Table S11) and prevented the age-related EDD decrease (Figure 3B). We have tested an independent RNAi line targeting different sequences of *dJun* and observed similar beneficial effects on HP, AI and EDD in 45 days old flies (Figure S5A). In addition, RNAi mediated inactivation of *kayak* (the Drosophila Fos homologue) also significantly improved AI and EDD in old flies (Figure S5A), confirming the central role of the Fos/Jun heterodimer AP1 in cardiac aging. We also analyzed the effect of *dJun* overexpression on heart function (dJun OE condition, Figure S5B). We observed an increased HP in 3 days old and 10 days old flies (respectively 23% and 10%). In older flies, *dJun* overexpression did not affect HP, probably because the JNK pathway was already activated.

**Table 3:** Genes downregulated at 40 days following RNAi mediated dJun knockdown.

	N° of genes(1)	GO term enrichment(2)	Cluster 2 Enrichment(3)	JNK pathway enrichment(4)	Predicted AP1 targets enrichment(5)
**Repressed**	1361	vesicle-mediated transport	138/635p = 10^−19^	29/135p = 1.1 10^−5^	23/99p = 1.8 10^−5^

Overview of genes repressed in 40 days old cardiac tubes following *dJun* knockdown. Enrichment p values were calculated using a test based on hypergeometric distribution.

(1) Deregulated genes between 40 days-old dissected cardiac tubes from *Hand-GS/UAS-dJun-IR* flies fed or not with 100 µg/ml RU486 were determined using Rank Product software from normalized expression data. Genes repressed in RU fed flies compared to the control non fed flies with a fold change of at least 1.5 fold between both conditions were retained for analysis (See Table S14).

(2) GO term enrichments were determined using the Flymine database (www.flymine.org) (see Table S15 for details).

(3) See Table 1 and Table S2 (induced genes at 40 days compared to 10 days).

(4) [17], [18].

(5) See Table S8.

JNK may be activated in the fly heart through OS signaling since ROS are potent activators of JNK by several mechanisms (reviewed [30]). However, JNK pathway target genes were not enriched following *cat* misexpression (1/133 genes, p = 0.53, see Table 2). Additional experiments are therefore required to determine the causes for JNK activation in aging hearts. The set of potential AP1 transcriptional targets that are up-regulated during aging is strongly enriched in genes involved in cytoskeleton organization and, in particular, in regulation of actin polymerization (Table S10). In addition, the set of genes that are deregulated following cardiac specific *dJun* knockdown at adulthood is enriched in genes involved in vesicle mediated transport (Table S15). This suggests that dJun activation in old flies may affect heart function by remodeling the actin network and/or by affecting vesicle mediated transport in cardiomyocytes; this notion should be explored in further studies. In mammals, the role of the JNK pathway in heart function and diseases (including cardiac hypertrophy, ischemia/reperfusion injury and pathological remodeling) is unclear and both *in vitro* and *in vivo* studies have given contradictory results (recently reviewed in [31]). Here, we show unambiguously that the age-dependent activation of Jra/dJUN in the fly heart is detrimental rather than protective. JNK has numerous evolutionarily conserved downstream targets besides the AP1 family members Jun and Fos, notably Forkhead Box O (FoxO) proteins [32], [33], [34]. Interestingly, overexpression of dFoxo prevents the decline in cardiac performance in aging flies [4]. Combined with our results, this suggests that, at least in Drosophila, JNK plays a complex and dual role in heart aging that can be decoupled: positive effects mediated by the insulin signaling pathway (IIS), and negative effects mediated by AP1.

Motifs associated with Vri/dNFIL3 were over-represented in genes down regulated with age. We confirmed that *vri* is able to regulate putative target genes identified by *cis*TargetX, by overexpressing *vri* and analyzing the expression of 3 of its putative targets by Q-PCR in 10 days old adults. As shown in Figure S6C, all 3 tested genes were repressed following *vri* overexpression, indicating that *vri* is a bona fide repressor of their expression. We therefore investigated its involvement in cardiac aging. Overexpression of *vri* (vrille OE condition) led to a large decrease in heart rate even by age 10 days. This effect was dependent on the level of Vri overexpression: 10 days old flies under RU486 10 µg/ml treatment did not exhibit increased HP, whereas under RU486 100 µg/ml treatment (the RU486 concentration currently used in this study), HP was increased by 38% compared to controls (Figure S6B). In 45 day-old flies, HP was 142% higher than in controls of the same age (Figure 3A and 3E, Video S8), whereas AI was not significantly different. This strong increase in HP was associated with a moderate dilated heart phenotype: between ages 10 and 45 days, EDD increased by 19% in flies overexpressing *vri*, whereas in control flies EDD decreased by 13%. Inversely, *vri* inactivation strongly improved cardiac performance in old flies (vrille-IR condition, Figure 3A, 3B, and 3D; Video S9 and Table S11). In control hearts, HP increased by 55% between ages 10 and 45 days, whereas it only increased by 6% when *vri* was inactivated. Inactivation of *vri* prevented EDD decrease (EDD remained stable between ages 10 and 45) and significantly reduced arrhythmia: AI was 23.6% and 36% lower in 10-day-old and 45-day-old flies than controls of the same ages. We have tested an independent RNAi line targeting different sequences of vrille (vrille-IR(II)) and also observed beneficial effects on AI, HP and EDD (Figure S6A).

These data clearly establish that Vri/dNFIL3 is a major regulator of heart senescence in flies. Furthermore, our molecular analyses suggest that it acts mainly by repressing downstream targets. The set of potential Vri/NFIL3 transcriptional targets that were down-regulated during the aging process was strongly enriched in genes encoding mitochondrial proteins (Table S10) and in particular those of the mitochondrial respiratory chain complexes. This transcriptional control has been confirmed by Q-PCR for CG11015, a component of the complex V of the electron transport chain (Figure S6C). This suggests that Vri may promote mitochondrial dysfunction in the aging heart through repression of genes encoding mitochondrial proteins. The effect of Vri on EDD is complex, since Vri inactivation prevented age-related EDD decrease, and Vri overexpression induced a dilated phenotype (increased EDD). Whether these two phenotypes involved the same mechanism or relied on independent Vri downstream targets remains to be clarified. Interestingly, in humans, mitochondrial respiratory chain disorders are frequently associated with hypertrophic or dilated cardiomyopathies (reviewed in [35]), suggesting that the dilated heart phenotype induced by Vri overexpression in flies may be due to strong mitochondrial respiratory defects.

We also observed a transcriptional repression of *prx5* by Vrille (Figure S6C). *prx5* encodes a peroxiredoxin localized in several cellular compartments including mitochondria, that confers resistance against oxidative stress and promotes longevity in Drosophila [36]. This demonstrates that Prx5 is a downstream target of Vri that may couple the Vri pathway with ROS levels during the cardiac aging process. Recently, NFIL3 has been shown to be required for correct zebrafish heart development [37]. However, the role of NFIL3 in adult heart function and aging has not been described, and investigating the role of NFIL3 in cardiac aging in mammals may be fruitful.

### Conclusion

Genetic redundancy and long lifespan make it difficult to investigate the genetic control of cardiac senescence in mammals. Our functional genomic approach in Drosophila allowed these problems to be avoided and led to the identification of key components of the gene regulatory network of cardiac aging.

First, we demonstrate that, as suggested in mammals, oxidative stress plays a central role in fly cardiac senescence. Our observations therefore support the idea that mechanisms of cardiac senescence are evolutionary conserved. Second, although the role of the JNK pathway in age-related processes in mammals is controversial, we show here that the dJun TF is activated during cardiac aging and has a detrimental effect on cardiac performance. Third, the transcription factor Vri/dNFIL3 is shown to play a central role in the cardiac aging process. Given that Drosophila heart shares lots of common points with that of vertebrates, our findings suggest that Vri and dJun orthologues are putative targets for counteracting cardiac aging in mammals.

To conclude, our work illustrates the relevance of data driven tissue-specific functional genomic analysis of the aging process in a model organism which allows straightforward genetic manipulations. Numerous studies, including transcriptome analysis in rodent models indicate that the effects of aging are in large part tissue-specific. Accordingly, half of the genes identified in our transcriptome analysis appear to be uniquely deregulated in the drosophila heart, suggesting cardiac specific mechanisms of aging. This is further supported by our observation that activation of the JNK/AP1 pathway is linked to accelerated heart senescence, while its activation organism-wide lead to increased life-span [38]. This probably indicates that activation of the JNK pathway has different outcomes with respects to senescence in different tissues or organs. As a matter of facts, its activation in the nervous system increases longevity [16], but reduces lifespan when achieved in intestinal stem cells [39]. Identification of the downstream effectors of this pathway in these different tissues and comparison with the cardiac specific targets identified in the present study may allow shedding light on its age related tissue specific effects.

## Materials and Methods

### Transcriptome analysis

Cardiac transcriptome dynamics between 10 and 40 days: Adult males (w1118, Canton S isogenic line) were grown in batches of 30 flies at 25°C and 60% hygrometry. Total RNA was extracted with Trizol and mRNA amplified using messageAmp (Ambion). Labeled aRNA was hybridized with Nimblegene arrays. After normalization of expression levels, differentially expressed genes were determined using the Limma software package. Gene Ontology enrichments were assessed using Flymine (www.flymine.org) and Transcription Regulatory Networks studied using *cis*TargetX (http://med.kuleuven.be/cme-mg/lng/cisTargetX/).

Cardiac transcriptome modifications induced by Catalase A Gain and Loss of Function: Adult males (*w;+/+; Hand-GS/UAS-Catalase-IR* (Cat knockdown) and *w;+/+; Hand-GS/UAS-Catalase* (Cat overexpression)) were grown in batches of 30 flies at 25°C and 60% hygrometry on food supplemented with 100 µg/ml of RU. RNA extraction was performed as above. Labeled aRNA were hybridized with Agilent arrays and differentially expressed genes determined using the Rank Product software within the TMev software suite.

Cardiac transcriptome modifications induced by dJun RNAi mediated knockdown: Adult males (*w;+/+; Hand-GS/UAS-dJun-IR)* were grown in batches of 30 flies at 25°C and 60% hygrometry on food supplemented or not with 100 µg/ml of RU. RNA extraction, micro array hybridization and data analysis were performed as described in the case of Catalase gain and loss of function.

### *In vivo* imaging of fly hearts

Flies expressing the GFP protein targeted to the mitochondria (mitoGFP) to label the cardiac tube were anaesthetized with Triethylamine and observed under a Zeiss SteREO Lumar.V12 Stereomicroscope, with a NeoLumar S 1.5× objective. Video movies were acquired with an AxioCamHR Camera. M-Modes were generated by horizontal alignment of rows extracted at the same position from each movie frame by using ImageJ, with automated positioning of the acquisition zone. Cardiograms (defined by the distance between the maxima of GFP fluorescence on each side of the median position of the heart at each time point) were then generated from M-Modes using an image processing algorithm developed with Matlab R2010b. The temporal positions for each end-systolic and end-diastolic position of the heart were extracted by finding all local maxima and minima on the cardiogram. The resulting file was incorporated into an Access DataBase to extract, for each cardiogram, the Heart Period (HP) and the Arrhythmicity Index (AI). Statistical significance was assessed by non-parametric Wilcoxon analysis [40].

Details of the Materials and Methods can be found in Text S1.

## Supporting Information

Figure S1 (Related to Table 1.) Q-PCR validation of microarray analysis. Seven genes (3 from cluster 1 (ATPSyn-Cf6, prx5 and CG11015) and 4 from cluster 2 (CG6981, rost, tsp42Ed and dJun)) were selected and their relative cardiac expression at 10 and 40 days was tested by RQ-PCR. RP49 was used as a reference endogenous gene for normalization. All cluster 1 genes displayed weaker expression at 40 days than to 10 days. By contrast, all cluster 2 genes were expressed at higher levels at 40 days.
(TIF)

Figure S2 Automated analysis of heartbeat. (A) The GeneSwitch protein (GS) is a modified GAL4 protein that recognizes and activates UAS-dependent transgenes only in the presence of RU486 (added to Drosophila food). In the system we used, the GS protein is expressed specifically in the heart under the control of the Hand Promoter (Hand-GS driver) and activates expression of a GFP protein targeted to mitochondria (mitoGFP) thereby labeling the heart. This driver can also be used to mediate overexpression or ds-RNA-mediated inactivation of candidate genes. (B) UV light, necessary to view the GFP fluorescence, moderately increased the mean Heart Rate by 19%. Heart Rates of *w/Y;UAS-mitoGFP/+* flies under visible light (−UV, n = 11) or UV (+UV, n = 16) were determined.*: p<5.10^−2^. M-Modes obtained under visible light (−UV) showed a rapid Heart Rate (HR) between 6,3 and 6,5 Hz interspersed with more prolonged beats, a pattern observed shortly after anesthesia. UV light suppressed these prolonged beats and stabilized the HR around 6,5 Hz in 10-day-old flies. (C) A row of pixels is selected to generate the M-mode. An algorithm developed with Matlab first finds the median position of the heart (red line) and then the local maxima on each side of this position (green plots). The algorithm finally generates a cardiogram defined by the distance between these two maxima over time (distance in pixels for each movie frame). See Text S1 for detailed description.
(TIF)

Figure S3 Heart performance is not affected by expression of luciferase or a control ds-RNA and is not dependent on RU concentration. (A) Heart Period (ms) and Arrhythmicity Index of *w/Y;UAS-mitoGFP/+; Hand-GS/+* (+), *w/Y;UAS-mitoGFP/+;Hand-GS/UAS-luciferase* (UAS-luci), and *w/Y;UAS-mitoGFP/UAS-sgs1-IR;Hand-GS/+* (UAS-sgs1-IR) male flies treated with RU 486 (100 µg/ml of food) during adulthood. Sgs1-IR was used as a control ds-RNA construct; *Sgs1* is a gene exclusively expressed in larval salivary gland. All values are means (±SEM). +/10 days: n = 26; +/30 days: n = 34; +/45 days: n = 34; +/60 days: n = 32; luci/10 days: n = 23; luci/30 days: n = 16; luci/45 days: n = 29; luci/60 days: n = 23; sgs1-IR/10 days: n = 7; sgs1-IR/30 days: n = 8; sgs1-IR/45 days: n = 9; sgs1-IR/60 days: n = 9. Luci and sgs1-IR flies did not exhibit significant differences (p<5.10^−2^) to control (+) flies of the same age, at any time point, except for a slightly lower AI in 10-day-old sgs1IR flies. (B) Heart Periods (ms) and Arrhythmicity Indexes of *w/Y;UAS-mitoGFP/+; Hand-GS/UAS-luciferase* (luci) male flies treated with 20 (RU 20) or 100 (RU100) µg of RU486 per ml of food during adulthood. All values are means (±SEM). RU 20/10 days: n = 8; RU 20/30 days: n = 9; RU 20/45 days: n = 5; RU 20/60 days: n = 6; RU 100/10 days: n = 23; RU 100/30 days: n = 16; RU 100/45 days: n = 29; RU 100/60 days: n = 23. There were no significant differences between RU 20 and RU 100 flies of the same age (p<5.10^−2^). (C) End Diastolic Diameters of 10-day-old and 60 day-old *w/Y;UAS-mitoGFP/+; Hand-GS/+* (+), *w/Y;UAS-mitoGFP/+;Hand-GS/UAS-luciferase* (UAS-luci), and *w/Y;UAS-mitoGFP/UAS-sgs1-IR;Hand-GS/+* (UAS-sgs1IR) male flies treated with RU 486 (100 µg/ml of food) during adulthood. All values are means (±SEM). +/10 days: n = 36; +/60 days: n = 41; luci/10 days: n = 15; luci/60 days: n = 11; sgs1-IR/10 days: n = 7; sgs1-IR/60 days: n = 9. Luci and sgs1IR flies did not exhibit significant differences to control (+) flies of the same age and presented a significant decrease in EDD between age 10 days and 60 days. * p<5.10^−2^, ** p<5.10^−3^.
(TIF)

Figure S4 Heart-specific expression of CatalaseB, but not SOD1, improves cardiac performance. Heart Period (ms) and Arrhythmicity Index of *w/Y;UAS-mitoGFP/+; Hand-GS/+* (+), *w/Y;UAS-mitoGFP/UAS-CatalaseB;Hand-GS/+* (CatB OE), *w/Y;UAS-mitoGFP/UAS-SOD1-IR;Hand-GS/+* (SOD1-IR) and *w/Y;UAS-mitoGFP/UAS-SOD1;Hand-GS/+* (SOD1 OE) male flies treated with RU 486 (100 µg/ml of food) during adulthood. All values are means (±SEM). (A) +/10 days: n = 26; +/30 days: n = 34; +/45 days: n = 34; +/60 days: n = 32; CatB OE/10 days: n = 17; CatB OE/30 days: n = 10; CatB OE/45 days: n = 19; CatB OE/60 days: n = 20. (B) +/10 days: n = 10; +/30 days: n = 10; +/45 days: n = 9; +/60 days: n = 8; SOD1-IR/10 days: n = 20; SOD1-IR/30 days: n = 9; SOD1-IR/45 days: n = 10; SOD1-IR/60 days: n = 4; (C) +/10 days: n = 10; +/30 days: n = 10; +/45 days: n = 9; +/60 days: n = 8; SOD1 OE/10 days: n = 9; SOD1 OE/30 days: n = 9; SOD1 OE/45 days: n = 8; SOD1 OE/60 days: n = 9. Significant differences with control (+) flies of the same age are indicated: * p<5.10^−3^.
(TIF)

Figure S5 Cardiac Performances in conditions of heart-specific AP1 inactivation and dJun overexpression. (A) Heart Period (HP), Arrhytmicity Index (AI) and End Diastolic Diameter (EDD) of 45-day-old *w/Y;UAS-mitoGFP/+; Hand-GS/+* (+), *w/Y;UAS-mitoGFP/+;Hand-GS/UAS-dJun-IR* (dJun-IR), *w-UAS-dJunIR (II)/Y;UAS-mitoGFP/+; Hand-GS/+*(dJun-IR (II)) and *w/Y;UAS-mitoGFP/ +;Hand-GS/UAS-kayak-IR* (Kayak-IR)male flies treated with RU 486 (10 µg/ml of food) during adulthood. Results are shown as ratios of mean values to mean values of controls flies (+) of the same age (±SEM). +: n = 27; dJun-IR: n = 9, dJun-IR (II): n = 11; kayak-IR: n = 9. (B) Heart Period (HP) and Arrhytmicity Index (AI) of *w/Y;UAS-mitoGFP/+; Hand-GS/+* (+) and *w/Y;UAS-mitoGFP/UAS-dJunOE; Hand-GS/+* (dJun OE) male flies treated with RU 486 (100 µg/ml of food) during adulthood. +/3 days: n = 13; +/10 days: n = 46; +/30 days: n = 34; dJun OE/3 days: n = 10; dJun OE/10 days: n = 40; dJun OE/30 days: n = 10. Significant differences with control (+) flies of the same age are indicated: * p<5.10^−2^, ** p<5.10^−3^.
(TIF)

Figure S6 Cardiac Performances in conditions of heart-specific Vri inactivation and overexpression and Validation of putative Vri targets. (A) Heart Period (HP), Arrhytmicity Index (AI) and End Diastolic Diameter (EDD) of *w/Y;UAS-mitoGFP/+; Hand-GS/+* (+) and *w/Y;UAS-mitoGFP/+;Hand-GS/UAS-vrille-IR(II)* (vrille-IR (II)) male flies treated with RU 486 (100 µg/ml of food) during adulthood. All values are means (±SEM). +/3 days: n = 13; +/10 days: n = 26; +/30 days: n = 34; +/45 days: n = 34; vrille-IR (II)/3 days: n = 9; vrille-IR (II)/10 days: n = 11; vrille-IR (II)/30 days: n = 19; vrille-IR (II)/45 days: n = 7. Significant differences with control (+) flies of the same age are indicated * p<5.10^−2^, ** p<5.10^−3^. (A) Heart Period (HP) of *w/Y;UAS-mitoGFP/+; Hand-GS/+* (+) and *w/Y;UAS-mitoGFP/+;Hand-GS/UAS-vrille OE* (vrille OE) male flies treated with 10 (RU10) or 100 (RU100) µg of RU486 per ml of food during adulthood. All values are means (±SEM). + RU10: n = 5; + RU100: n = 10; vrille OE RU10: n = 7; vrille OE RU100: n = 10. Significant differences are indicated * p<5.10^−2^, ** p<5.10^−3^. (B) 3 genes from cluster 1 (*ATPsyn-Cf6* (CG4412), *prx5*(CG7217) and *CG11015*, encoding a component of the complex V of the mitochondrial respiratory chain) predicted by *cis*targetX as potential Vri targets were tested by RQ-PCR following *vri* overexpression (UAS>Vri) using the inducible driver Da>GeneSwitch. Relative expression between induced (+RU) and non-induced (−RU) was measured. RP49 was used as a reference endogenous gene for normalization. All 3 genes displayed weaker expression following *vri* overexpression.
(TIF)

Table S1 (Related to Table 1.) Differentially expressed probes. All probes showing statistically significant differential expression between 10 and 40 day-old flies are listed. Probes ID (“reporterID”), gene symbol (transcript) and FlyBase unique identifier (FBGN) are indicated. Several FlyBase identifiers correspond to annotations withdrawn from the latest version of FlyBase and are indicated as gray cells at the end of the spreadsheet. For each probe, the mean of signal intensity is indicated (AveExpre), together with the log2 value of the fold change [log2 FC (10d/40d)] and the adjusted P value, as calculated using limma software (see Experimental procedures). Clusters 1 and 2 correspond to probes repressed and induced, respectively, at 40 days of age relative to 10 days of age.
(XLSX)

Table S2 (Related to Table 1.) Differentially expressed genes. Unique differentially expressed genes between 10 days and 40 days are listed. FlyBase unique identifier (FB ID), gene symbol and computer gene numbers (annotation ID) are indicated. Seven FlyBase identifiers correspond to annotations withdrawn from the latest version of FlyBase and are indicated as gray cells at the end of the spreadsheet. For each gene, the mean signal intensity is indicated (<signal>), together with the mean value of the fold change (<FC>) and the corrected P value, as calculated using limma software (see Experimental procedure). Clusters 1 and 2 correspond to genes repressed and induced, respectively, at 40 days of age relative to 10 days of age.
(XLSX)

Table S3 (Related to Table 1.) Comparison of aging heart transcriptome with age-related transcriptional changes in different body parts. Differentially expressed cardiac genes at 10 and 40 days were compared to genes differentially expressed in whole body and other body parts at similar ages. In all cases, cluster 1 (green) includes genes repressed in aged tissues and cluster 2 (red) genes induced in aged tissues. For each gene differentially expressed in the cardiac tube, the FBgn (FB), symbol (symbol) CG number (CG), the mean of the fold change (<FC>) and corresponding cluster (cluster) is indicated. Aging 1: analysis performed in [10]. Aging-WB, aging-Thorax, Aging-Head correspond to differential transcriptome analyses performed with young and aged drosophila, respectively, investigating whole body, thorax and head and are described in [9].
(XLSX)

Table S4 (Related to Table 1.) Biological processes over-represented among differentially expressed genes. Gene Ontology terms and Pathway enrichment in differentially expressed genes were identified using Flymine. Gene ontology (GO), Pathway ID and names are indicated, together with the calculated P-value and associated genes in corresponding clusters.
(XLSX)

Table S5 (Related to Table 1 and Table S3.) Deregulated genes involved in mitochondrial bioenergetics (cluster 1), vesicle-mediated transport and defense/immune/stress response (cluster 2). Genes associated with corresponding GO or pathway terms are listed, together with the mean of the fold change (<FC>).
(XLSX)

Table S6 (Related to Table 1.) Comparison of the set of up-regulated genes with MnSOD responsive genes. MnSOD-dependent genes identified in MnSOD-overexpressing flies [13] and presenting transcriptional modifications in the aging heart are listed. Cluster indexes identify either repressed genes (cluster = 1) or induced genes (cluster = 2). There is significant overlap (p<10^−20^ see Table 1) between the two studies among the 147 genes over-expressed in both conditions. In contrast, no enrichment is observed for down-regulated genes.
(XLSX)

Table S7 (Related to Table 1.) Comparison of the set of up-regulated genes with paraquat -responsive genes. Oxidative stress-dependent genes identified in paraquat-treated flies [14] and presenting transcriptional modifications in the aging heart are listed. Cluster indexes identify either repressed genes (cluster = 1) or induced genes (cluster = 2). There was significant overlap (p<2 10^−5^ see Table 1) between the 64 genes overexpressed in the two conditions. In contrast no enrichment was observed for downregulated genes.
(XLSX)

Table S8 (Related to Table 1.) *cis*TargetX analysis and prediction of putative Vri and AP1 target genes. Cluster 1 and 2 gene sets were separately subjected to *cis*TargetX analysis with a z score cutoff set at 2.5. The motifs and corresponding enrichment scores (z score), as provided by the web server, are given for each case. Putative target genes for dNFIL3, AP1 and dEvi-1 are indicated for the most over-represented motif in each case (using the “candidate target” function of the web server, which provides the significantly highly ranked genes for a selected motif).
(XLSX)

Table S9 (Related to Table 1.) Comparison of the set of up-regulated genes with JNK-pathway-responsive genes. JNK pathway-dependent genes modulated following innate immune challenge were identified in two studies on Drosophila S2 cells [17], [18]. Cluster 2 genes also found among Hemipterous (hep, the JNK MAP kinase-Kinase)-dependent genes = and/or Basket (bsk, the JNK MAP kinase)-dependent genes  = following Lipo-polysaccharide challenge are listed. Hep- and Bsk-dependent genes represent a total of 133 non redundant genes, of which 29 are present in cluster 2 (p<10^−7^ see Table 1).
(XLSX)

Table S10 (Related to Table 1.) Over-represented biological processes in candidate Vri and AP1 target genes. Gene Ontology terms and Pathway enrichment in putative dNFIL3 and AP1 target genes (from cluster 1 and 2, respectively, see table S2) were identified using Flymine. Gene ontology (GO) and Pathway ID and names are indicated, together with the calculated P-value and associated genes in the corresponding clusters.
(XLSX)

Table S11 (Related to Figure 2, Figure 3, Figure S3, Figure S4.) Analysis of evolution of heart parameters as a function of age. For each different couple of conditions related to Figure 2, Figure 3, and Figures S3 and S4 (columns 2 and 3), we performed a linear fit of the data and analyzed the statistical significance between the slope of the two curves by the method of Zar [40]. The slopes of the fitted curves for the heart period and the P-value for the null hypothesis of similar slopes are reported in columns 4, 5 and 6 respectively. Similar data is reported in columns 7, 8 and 9 for the analysis of the Arrhythmicity Index. P-values corresponding to significant differences between the slopes are outlined in grey. To allow comparisons between all the experiments, the data presented here correspond to analysis performed for ages greater or equal to 10 days. When data for 3 day old flies were available (Figure 3C and 3E) additional analysis were performed and provided similar conclusions for the significance of slope differences.
(XLSX)

Table S12 (Related to Table 2.) Genes differentially expressed between Catalase knockdown and Gain of Function. Differentially expressed genes between HandGS/UAS>Cat-IR and HandGS/UAS>Cat cardiac tubes from 40 days old flies fed on RU486 containing food are listed. FlyBase unique identifier (FB ID), gene symbol and computer gene numbers (annotation ID) are indicated. For each gene, the mean value of the fold change (<FC>) and the corrected P value, as calculated using Rank Product software (see Experimental procedure) are indicated. Clusters of genes induced or repressed in Catalase knockdown compared to Gain of Function are shown.
(XLSX)

Table S13 (Related to Table 2.) Biological processes over-represented among genes differentially expressed between Catalase knockdown and Gain of Function. Gene Ontology terms in differentially expressed genes were identified using Flymine. Gene ontology (GO) ID and name are indicated, together with the calculated P-value and associated genes in corresponding clusters.
(XLSX)

Table S14 (Related to Table 3.) Genes repressed upon dJun knockdown. Genes down regulated in HandGS/UAS>dJun-IR cardiac tubes from 40 days old flies fed on RU486 containing food compared to cardiac tubes of the same genotype and age fed on food not containing RU486 are listed. FlyBase unique identifier (FB ID), gene symbol and computer gene numbers (annotation ID) are indicated. For each gene, the mean value of the fold change (<FC>) and the corrected P value, as calculated using Rank Product software (see Experimental procedure) are indicated.
(XLSX)

Table S15 (Related to Table 3.) Biological processes over-represented among genes repressed upon dJun knockdown. Gene Ontology terms in differentially expressed genes were identified using Flymine. Gene ontology (GO) ID and name are indicated, together with the calculated P-value and associated genes in corresponding clusters.
(XLSX)

Text S1 Detailed Materials and Methods.
(DOCX)

Video S1 Heart beats of a 10-day-old fly. The movie is acquired on a 10-day-old *w/Y;UAS-mitoGFP/+; Hand-GS/+* (+) male fly. The speed of the movie is 10 fps (Frames per second).
(AVI)

Video S2 Heart beats of a 45-day-old fly. The movie is acquired on a 45-day-old *w/Y;UAS-mitoGFP/+; Hand-GS/+* (+) male fly. The speed of the movie is 10 fps.
(AVI)

Video S3 Heart beats of a 60-day-old fly. The movie is acquired on a 60-day-old *w/Y;UAS-mitoGFP/+; Hand-GS/+* (+) male fly. The speed of the movie is 10 fps.
(AVI)

Video S4 Heart beats of a 60-day-old Cat-IR fly. The movie is acquired on a 60-day-old *w/Y;UAS-mitoGFP/+; Hand-GS/UAS-Catalase-IR* (Cat-IR) male fly. The speed of the movie is 10 fps.
(AVI)

Video S5 Heart beats of a 60-day-old Cat OE fly. The movie is acquired on a 60-day-old *w/Y;UAS-mitoGFP/UAS-Catalase* male fly. The speed of the movie is 10 fps.
(AVI)

Video S6 Heart beats of a 60-day-old fly treated with EUK-8. The movie is acquired on a *w/Y;UAS-mitoGFP/+; Hand-GS/+* male fly treated with EUK-8 (0.2 mM) from the age of 30 days. The speed of the movie is 10 fps.
(AVI)

Video S7 Heart beats of a 45-day-old dJun-IR fly. The movie is acquired on a 45-day-old *w/Y;UAS-mitoGFP/+;Hand-GS/UAS-dJun-IR* (dJun-IR) male fly. The speed of the movie is 10 fps.
(AVI)

Video S8 Heart beats of a 45-day-old vrille OE fly. The movie is acquired on a 45-day-old *w/Y;UAS-mitoGFP/UAS-vrille; Hand-GS/+* (vrille OE) male fly. The speed of the movie is 10 fps.
(AVI)

Video S9 Heart beats of a 45-day-old vrille-IR fly. The movie is acquired on a 45-day-old */Y;UAS-mitoGFP/UAS-vrille-IR; Hand-GS/+* (vrille-IR) male fly. The speed of the movie is 10 fps.
(AVI)

## References

[1] Lakatta EG (2001) Heart aging: a fly in the ointment? Circ Res 88: 984–986. 11375266 10.1161/hh1001.091963

[2] Occor K, Perrin L, Lim H Y, Quian L, Bodmer R (2007) Genetic control of heart function and aging in Drosophila. Trends Cardiovasc Med 17 5: 177–182. 17574126 10.1016/j.tcm.2007.04.001 PMC1950717

[3] Paternostro G, Vignola C, Bartsch DU, Omens JH, McCulloch AD, et al (2001) Age-associated cardiac dysfunction in Drosophila melanogaster. Circ Res 88: 1053–1058. 11375275 10.1161/hh1001.090857

[4] Wessells RJ, Fitzgerald E, Cypser JR, Tatar M, Bodmer R (2004) Insulin regulation of heart function in aging fruit flies. Nat Genet 36: 1275–1281. 15565107 10.1038/ng1476

[5] Rincon M, Rudin E, Barzilai N (2005) The insulin/IGF-1 signaling in mammals and its relevance to human longevity. Exp Gerontol 40: 873–877. 16168602 10.1016/j.exger.2005.06.014

[6] Akasaka T, Klinedinst S, Ocorr K, Bustamante EL, Kim SK, et al (2006) The ATP-sensitive potassium (KATP) channel-encoded dSUR gene is required for Drosophila heart function and is regulated by tinman. Proc Natl Acad Sci U S A 103: 11999–12004. 16882722 10.1073/pnas.0603098103 PMC1567687

[7] Aerts S, Quan XJ, Claeys A, Naval Sanchez M, Tate P, et al (2010) Robust target gene discovery through transcriptome perturbations and genome-wide enhancer predictions in Drosophila uncovers a regulatory basis for sensory specification. PLoS Biol 8: e1000435 doi:10.1371/journal.pbio.1000435 20668662 10.1371/journal.pbio.1000435 PMC2910651

[8] Zahn JM, Sonu R, Vogel H, Crane E, Mazan-Mamczarz K, et al (2006) Transcriptional profiling of aging in human muscle reveals a common aging signature. PLoS Genet 2: e115 doi:10.1371/journal.pgen.0020115 16789832 10.1371/journal.pgen.0020115 PMC1513263

[9] Girardot F, Lasbleiz C, Monnier V, Tricoire H (2006) Specific age-related signatures in Drosophila body parts transcriptome. BMC Genomics 7: 69. 16584578 10.1186/1471-2164-7-69 PMC1481561

[10] Landis GN, Abdueva D, Skvortsov D, Yang J, Rabin BE, et al (2004) Similar gene expression patterns characterize aging and oxidative stress in Drosophila melanogaster. Proc Natl Acad Sci U S A 101: 7663–7668. 15136717 10.1073/pnas.0307605101 PMC419663

[11] Brink TC, Demetrius L, Lehrach H, Adjaye J (2009) Age-related transcriptional changes in gene expression in different organs of mice support the metabolic stability theory of aging. Biogerontology 10: 549–564. 19031007 10.1007/s10522-008-9197-8 PMC2730443

[12] Muller FL, Lustgarten MS, Jang Y, Richardson A, Van Remmen H (2007) Trends in oxidative aging theories. Free Radic Biol Med 43: 477–503. 17640558 10.1016/j.freeradbiomed.2007.03.034

[13] Curtis C, Landis GN, Folk D, Wehr NB, Hoe N, et al (2007) Transcriptional profiling of MnSOD-mediated lifespan extension in Drosophila reveals a species-general network of aging and metabolic genes. Genome Biol 8: R262. 18067683 10.1186/gb-2007-8-12-r262 PMC2246264

[14] Girardot F, Monnier V, Tricoire H (2004) Genome wide analysis of common and specific stress responses in adult drosophila melanogaster. BMC Genomics 5: 74. 15458575 10.1186/1471-2164-5-74 PMC526417

[15] Potier D, Atak ZK, Sanchez MN, Herrmann C, Aerts S (2012) Using cisTargetX to predict transcriptional targets and networks in Drosophila. Methods Mol Biol 786: 291–314. 21938634 10.1007/978-1-61779-292-2_18

[16] Wang MC, Bohmann D, Jasper H (2003) JNK signaling confers tolerance to oxidative stress and extends lifespan in Drosophila. Dev Cell 5: 811–816. 14602080 10.1016/s1534-5807(03)00323-x

[17] Boutros M, Agaisse H, Perrimon N (2002) Sequential activation of signaling pathways during innate immune responses in Drosophila. Dev Cell 3: 711–722. 12431377 10.1016/s1534-5807(02)00325-8

[18] Kim T, Yoon J, Cho H, Lee WB, Kim J, et al (2005) Downregulation of lipopolysaccharide response in Drosophila by negative crosstalk between the AP1 and NF-kappaB signaling modules. Nat Immunol 6: 211–218. 15640802 10.1038/ni1159

[19] Moore AW, Jan LY, Jan YN (2002) hamlet, a binary genetic switch between single- and multiple- dendrite neuron morphology. Science 297: 1355–1358. 12193790 10.1126/science.1072387

[20] Cowell IG (2002) E4BP4/NFIL3, a PAR-related bZIP factor with many roles. Bioessays 24: 1023–1029. 12386933 10.1002/bies.10176

[21] Roman G, Endo K, Zong L, Davis RL (2001) P[Switch], a system for spatial and temporal control of gene expression in Drosophila melanogaster. Proc Natl Acad Sci U S A 98: 12602–12607. 11675496 10.1073/pnas.221303998 PMC60100

[22] Choma MA, Izatt SD, Wessells RJ, Bodmer R, Izatt JA (2006) Images in cardiovascular medicine: in vivo imaging of the adult Drosophila melanogaster heart with real-time optical coherence tomography. Circulation 114: e35–36. 16831991 10.1161/CIRCULATIONAHA.105.593541

[23] Feala JD, Omens JH, Paternostro G, McCulloch AD (2008) Discovering regulators of the Drosophila cardiac hypoxia response using automated phenotyping technology. Ann N Y Acad Sci 1123: 169–177. 18375589 10.1196/annals.1420.019

[24] Yu L, Lee T, Lin N, Wolf MJ (2010) Affecting Rhomboid-3 function causes a dilated heart in adult Drosophila. PLoS Genet 6: e1000969 doi:10.1371/journal.pgen.1000969 20523889 10.1371/journal.pgen.1000969 PMC2877733

[25] Fink M, Callol-Massot C, Chu A, Ruiz-Lozano P, Izpisua Belmonte JC, et al (2009) A new method for detection and quantification of heartbeat parameters in Drosophila, zebrafish, and embryonic mouse hearts. Biotechniques 46: 101–113. 19317655 10.2144/000113078 PMC2855226

[26] Dai DF, Santana LF, Vermulst M, Tomazela DM, Emond MJ, et al (2009) Overexpression of catalase targeted to mitochondria attenuates murine cardiac aging. Circulation 119: 2789–2797. 19451351 10.1161/CIRCULATIONAHA.108.822403 PMC2858759

[27] Tanguy S, Boucher FR, Malfroy B, de Leiris JG (1996) Free radicals in reperfusion-induced arrhythmias: study with EUK 8, a novel nonprotein catalytic antioxidant. Free Radic Biol Med 21: 945–954. 8937880 10.1016/s0891-5849(96)00231-6

[28] Morten KJ, Ackrell BA, Melov S (2006) Mitochondrial reactive oxygen species in mice lacking superoxide dismutase 2: attenuation via antioxidant treatment. J Biol Chem 281: 3354–3359. 16326710 10.1074/jbc.M509261200

[29] Kawakami S, Matsuda A, Sunagawa T, Noda Y, Kaneko T, et al (2009) Antioxidant, EUK-8, prevents murine dilated cardiomyopathy. Circ J 73: 2125–2134. 19749480 10.1253/circj.cj-09-0204

[30] Matsuzawa A, Ichijo H (2008) Redox control of cell fate by MAP kinase: physiological roles of ASK1-MAP kinase pathway in stress signaling. Biochim Biophys Acta 1780: 1325–1336. 18206122 10.1016/j.bbagen.2007.12.011

[31] Rose BA, Force T, Wang Y (2010) Mitogen-activated protein kinase signaling in the heart: angels versus demons in a heart-breaking tale. Physiol Rev 90: 1507–1546. 20959622 10.1152/physrev.00054.2009 PMC3808831

[32] Essers MA, Weijzen S, de Vries-Smits AM, Saarloos I, de Ruiter ND, et al (2004) FOXO transcription factor activation by oxidative stress mediated by the small GTPase Ral and JNK. EMBO J 23: 4802–4812. 15538382 10.1038/sj.emboj.7600476 PMC535088

[33] Oh SW, Mukhopadhyay A, Svrzikapa N, Jiang F, Davis RJ, et al (2005) JNK regulates lifespan in Caenorhabditis elegans by modulating nuclear translocation of forkhead transcription factor/DAF-16. Proc Natl Acad Sci U S A 102: 4494–4499. 15767565 10.1073/pnas.0500749102 PMC555525

[34] Wang MC, Bohmann D, Jasper H (2005) JNK extends life span and limits growth by antagonizing cellular and organism-wide responses to insulin signaling. Cell 121: 115–125. 15820683 10.1016/j.cell.2005.02.030

[35] Berardo A, Musumeci O, Toscano A (2011) Cardiological manifestations of mitochondrial respiratory chain disorders. Acta Myol 30: 9–15. 21842587 PMC3185833

[36] Radyuk SN, Michalak K, Klichko VI, Benes J, Rebrin I, et al (2009) Peroxiredoxin 5 confers protection against oxidative stress and apoptosis and also promotes longevity in Drosophila. Biochem J 419: 437–445. 19128239 10.1042/BJ20082003 PMC2842572

[37] Weng YJ, Hsieh DJ, Kuo WW, Lai TY, Hsu HH, et al (2010) E4BP4 is a cardiac survival factor and essential for embryonic heart development. Mol Cell Biochem 340: 187–194. 20186462 10.1007/s11010-010-0417-6

[38] Biteau B, Karpac J, Hwangbo D, Jasper H (2011) Regulation of Drosophila lifespan by JNK signaling. Exp Gerontol 46: 349–354. 21111799 10.1016/j.exger.2010.11.003 PMC3079798

[39] Biteau B, Hochmuth CE, Jasper H (2008) JNK activity in somatic stem cells causes loss of tissue homeostasis in the aging Drosophila gut. Cell Stem Cell 3: 442–455. 18940735 10.1016/j.stem.2008.07.024 PMC3225008

[40] Zar J (1984). Biostatistical Analysis. 2nd ed: Prentice-Hall.

