# Supplementary figures and images for: dJun and Vri/dNFIL3 Are Major Regulators of Cardiac Aging in Drosophila
Source: PLoS Genet. 2012 Nov 29;8(11):e1003081. doi: 10.1371/journal.pgen.1003081 (PMC3510041; doi:10.1371/journal.pgen.1003081)

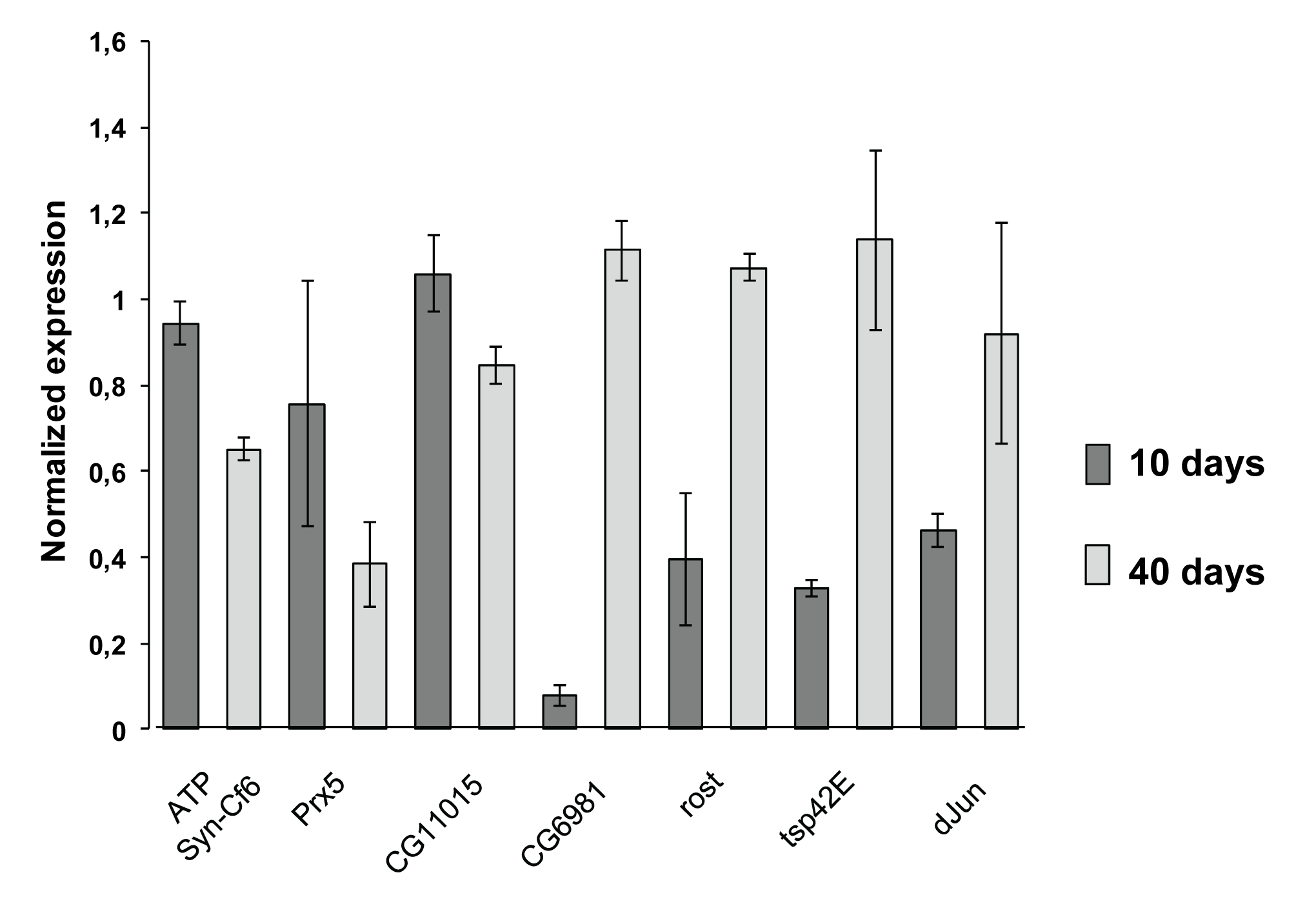

Supplement: Figure S1 — (Related to Table 1.) Q-PCR validation of microarray analysis. Seven genes (3 from cluster 1 (ATPSyn-Cf6, prx5 and CG11015) and 4 from cluster 2 (CG6981, rost, tsp42Ed and dJun)) were selected and their relative cardiac expression at 10 and 40 days was tested by RQ-PCR. RP49 was used as a reference endogenous gene for normalization. All cluster 1 genes displayed weaker expression at 40 days than to 10 days. By contrast, all cluster 2 genes were expressed at higher levels at 40 days. (TIF) [file pgen.1003081.s001.tif]

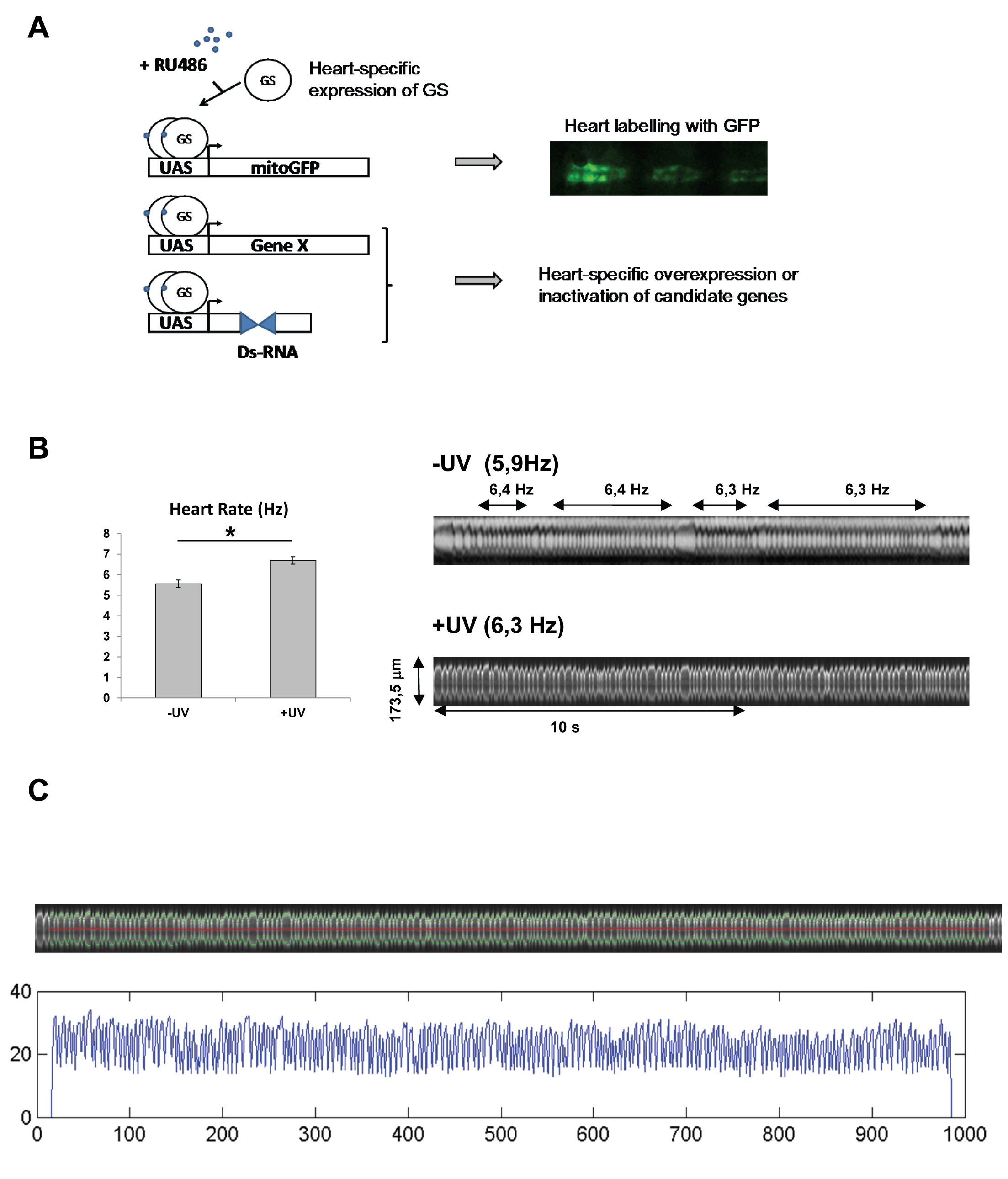

Supplement: Figure S2 — Automated analysis of heartbeat. (A) The GeneSwitch protein (GS) is a modified GAL4 protein that recognizes and activates UAS-dependent transgenes only in the presence of RU486 (added to Drosophila food). In the system we used, the GS protein is expressed specifically in the heart under the control of the Hand Promoter (Hand-GS driver) and activates expression of a GFP protein targeted to mitochondria (mitoGFP) thereby labeling the heart. This driver can also be used to mediate overexpression or ds-RNA-mediated inactivation of candidate genes. (B) UV light, necessary to view the GFP fluorescence, moderately increased the mean Heart Rate by 19%. Heart Rates of w/Y;UAS-mitoGFP/+ flies under visible light (−UV, n = 11) or UV (+UV, n = 16) were determined.*: p<5.10−2. M-Modes obtained under visible light (−UV) showed a rapid Heart Rate (HR) between 6,3 and 6,5 Hz interspersed with more prolonged beats, a pattern observed shortly after anesthesia. UV light suppressed these prolonged beats and stabilized the HR around 6,5 Hz in 10-day-old flies. (C) A row of pixels is selected to generate the M-mode. An algorithm developed with Matlab first finds the median position of the heart (red line) and then the local maxima on each side of this position (green plots). The algorithm finally generates a cardiogram defined by the distance between these two maxima over time (distance in pixels for each movie frame). See Text S1 for detailed description. (TIF) [file pgen.1003081.s002.tif]

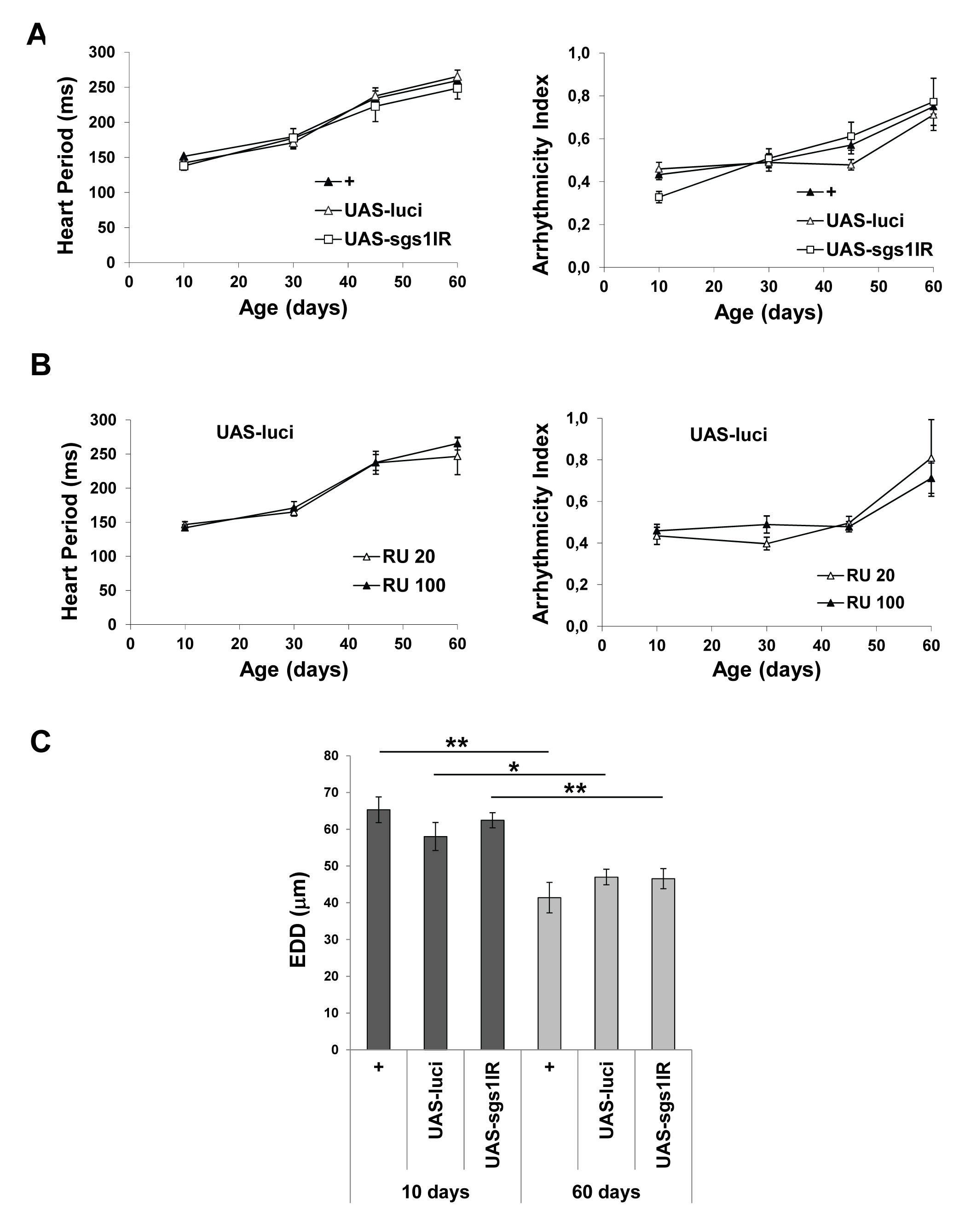

Supplement: Figure S3 — Heart performance is not affected by expression of luciferase or a control ds-RNA and is not dependent on RU concentration. (A) Heart Period (ms) and Arrhythmicity Index of w/Y;UAS-mitoGFP/+; Hand-GS/+ (+), w/Y;UAS-mitoGFP/+;Hand-GS/UAS-luciferase (UAS-luci), and w/Y;UAS-mitoGFP/UAS-sgs1-IR;Hand-GS/+ (UAS-sgs1-IR) male flies treated with RU 486 (100 µg/ml of food) during adulthood. Sgs1-IR was used as a control ds-RNA construct; Sgs1 is a gene exclusively expressed in larval salivary gland. All values are means (±SEM). +/10 days: n = 26; +/30 days: n = 34; +/45 days: n = 34; +/60 days: n = 32; luci/10 days: n = 23; luci/30 days: n = 16; luci/45 days: n = 29; luci/60 days: n = 23; sgs1-IR/10 days: n = 7; sgs1-IR/30 days: n = 8; sgs1-IR/45 days: n = 9; sgs1-IR/60 days: n = 9. Luci and sgs1-IR flies did not exhibit significant differences (p<5.10−2) to control (+) flies of the same age, at any time point, except for a slightly lower AI in 10-day-old sgs1IR flies. (B) Heart Periods (ms) and Arrhythmicity Indexes of w/Y;UAS-mitoGFP/+; Hand-GS/UAS-luciferase (luci) male flies treated with 20 (RU 20) or 100 (RU100) µg of RU486 per ml of food during adulthood. All values are means (±SEM). RU 20/10 days: n = 8; RU 20/30 days: n = 9; RU 20/45 days: n = 5; RU 20/60 days: n = 6; RU 100/10 days: n = 23; RU 100/30 days: n = 16; RU 100/45 days: n = 29; RU 100/60 days: n = 23. There were no significant differences between RU 20 and RU 100 flies of the same age (p<5.10−2). (C) End Diastolic Diameters of 10-day-old and 60 day-old w/Y;UAS-mitoGFP/+; Hand-GS/+ (+), w/Y;UAS-mitoGFP/+;Hand-GS/UAS-luciferase (UAS-luci), and w/Y;UAS-mitoGFP/UAS-sgs1-IR;Hand-GS/+ (UAS-sgs1IR) male flies treated with RU 486 (100 µg/ml of food) during adulthood. All values are means (±SEM). +/10 days: n = 36; +/60 days: n = 41; luci/10 days: n = 15; luci/60 days: n = 11; sgs1-IR/10 days: n = 7; sgs1-IR/60 days: n = 9. Luci and sgs1IR flies did not exhibit significant differences to control (+) flies of the sa [file pgen.1003081.s003.tif]

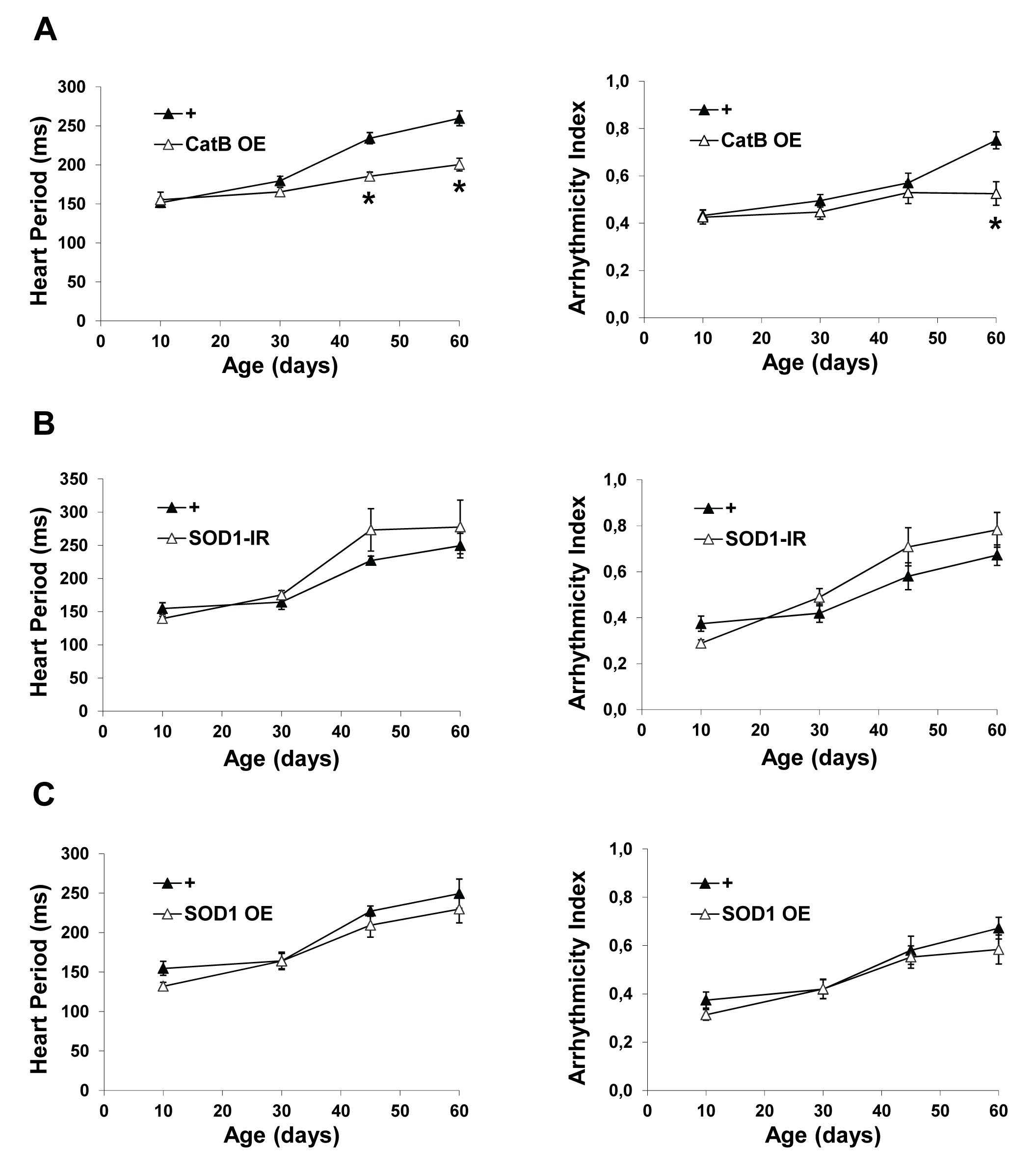

Supplement: Figure S4 — Heart-specific expression of CatalaseB, but not SOD1, improves cardiac performance. Heart Period (ms) and Arrhythmicity Index of w/Y;UAS-mitoGFP/+; Hand-GS/+ (+), w/Y;UAS-mitoGFP/UAS-CatalaseB;Hand-GS/+ (CatB OE), w/Y;UAS-mitoGFP/UAS-SOD1-IR;Hand-GS/+ (SOD1-IR) and w/Y;UAS-mitoGFP/UAS-SOD1;Hand-GS/+ (SOD1 OE) male flies treated with RU 486 (100 µg/ml of food) during adulthood. All values are means (±SEM). (A) +/10 days: n = 26; +/30 days: n = 34; +/45 days: n = 34; +/60 days: n = 32; CatB OE/10 days: n = 17; CatB OE/30 days: n = 10; CatB OE/45 days: n = 19; CatB OE/60 days: n = 20. (B) +/10 days: n = 10; +/30 days: n = 10; +/45 days: n = 9; +/60 days: n = 8; SOD1-IR/10 days: n = 20; SOD1-IR/30 days: n = 9; SOD1-IR/45 days: n = 10; SOD1-IR/60 days: n = 4; (C) +/10 days: n = 10; +/30 days: n = 10; +/45 days: n = 9; +/60 days: n = 8; SOD1 OE/10 days: n = 9; SOD1 OE/30 days: n = 9; SOD1 OE/45 days: n = 8; SOD1 OE/60 days: n = 9. Significant differences with control (+) flies of the same age are indicated: * p<5.10−3. (TIF) [file pgen.1003081.s004.tif]

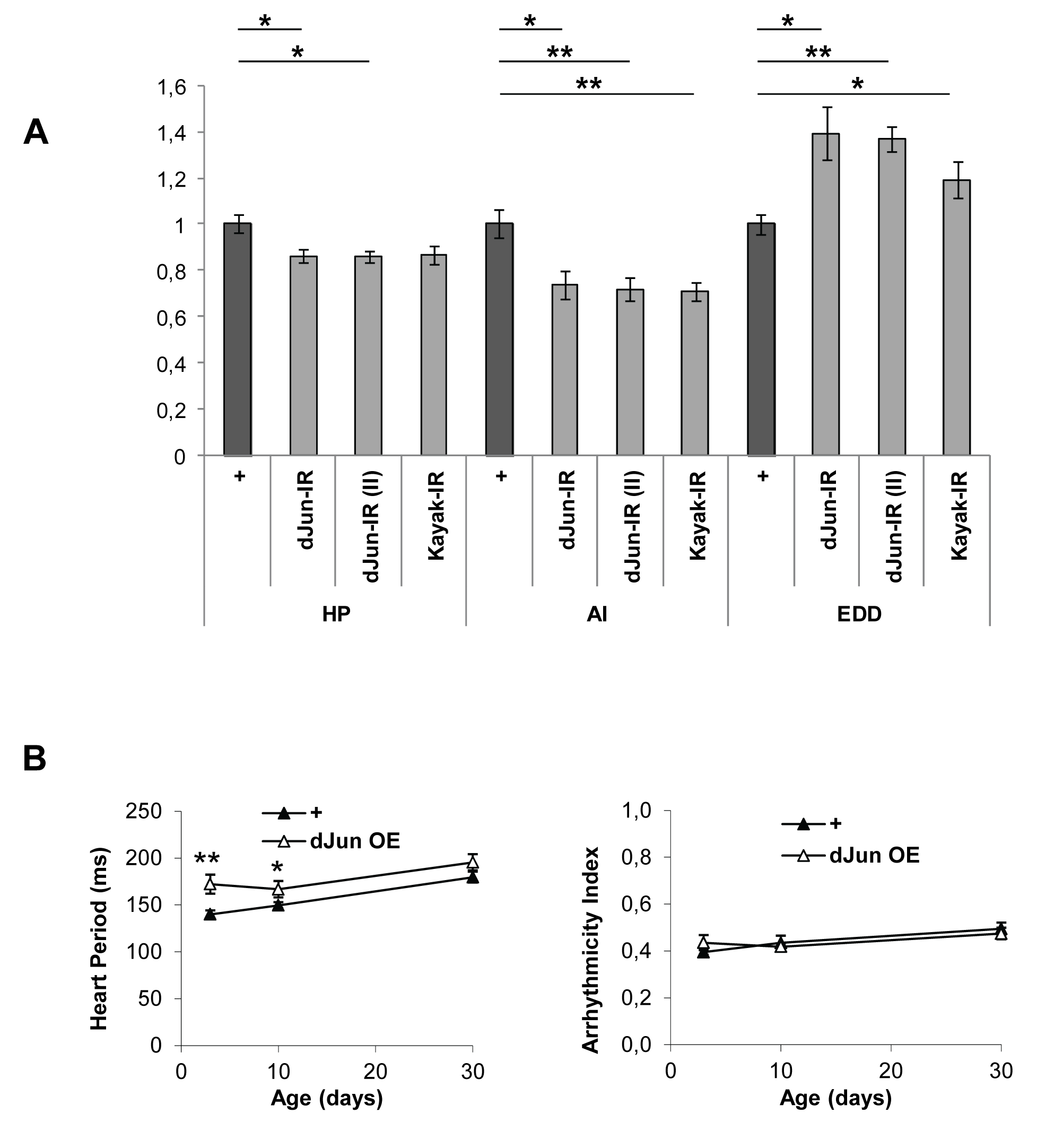

Supplement: Figure S5 — Cardiac Performances in conditions of heart-specific AP1 inactivation and dJun overexpression. (A) Heart Period (HP), Arrhytmicity Index (AI) and End Diastolic Diameter (EDD) of 45-day-old w/Y;UAS-mitoGFP/+; Hand-GS/+ (+), w/Y;UAS-mitoGFP/+;Hand-GS/UAS-dJun-IR (dJun-IR), w-UAS-dJunIR (II)/Y;UAS-mitoGFP/+; Hand-GS/+(dJun-IR (II)) and w/Y;UAS-mitoGFP/ +;Hand-GS/UAS-kayak-IR (Kayak-IR)male flies treated with RU 486 (10 µg/ml of food) during adulthood. Results are shown as ratios of mean values to mean values of controls flies (+) of the same age (±SEM). +: n = 27; dJun-IR: n = 9, dJun-IR (II): n = 11; kayak-IR: n = 9. (B) Heart Period (HP) and Arrhytmicity Index (AI) of w/Y;UAS-mitoGFP/+; Hand-GS/+ (+) and w/Y;UAS-mitoGFP/UAS-dJunOE; Hand-GS/+ (dJun OE) male flies treated with RU 486 (100 µg/ml of food) during adulthood. +/3 days: n = 13; +/10 days: n = 46; +/30 days: n = 34; dJun OE/3 days: n = 10; dJun OE/10 days: n = 40; dJun OE/30 days: n = 10. Significant differences with control (+) flies of the same age are indicated: * p<5.10−2, ** p<5.10−3. (TIF) [file pgen.1003081.s005.tif]

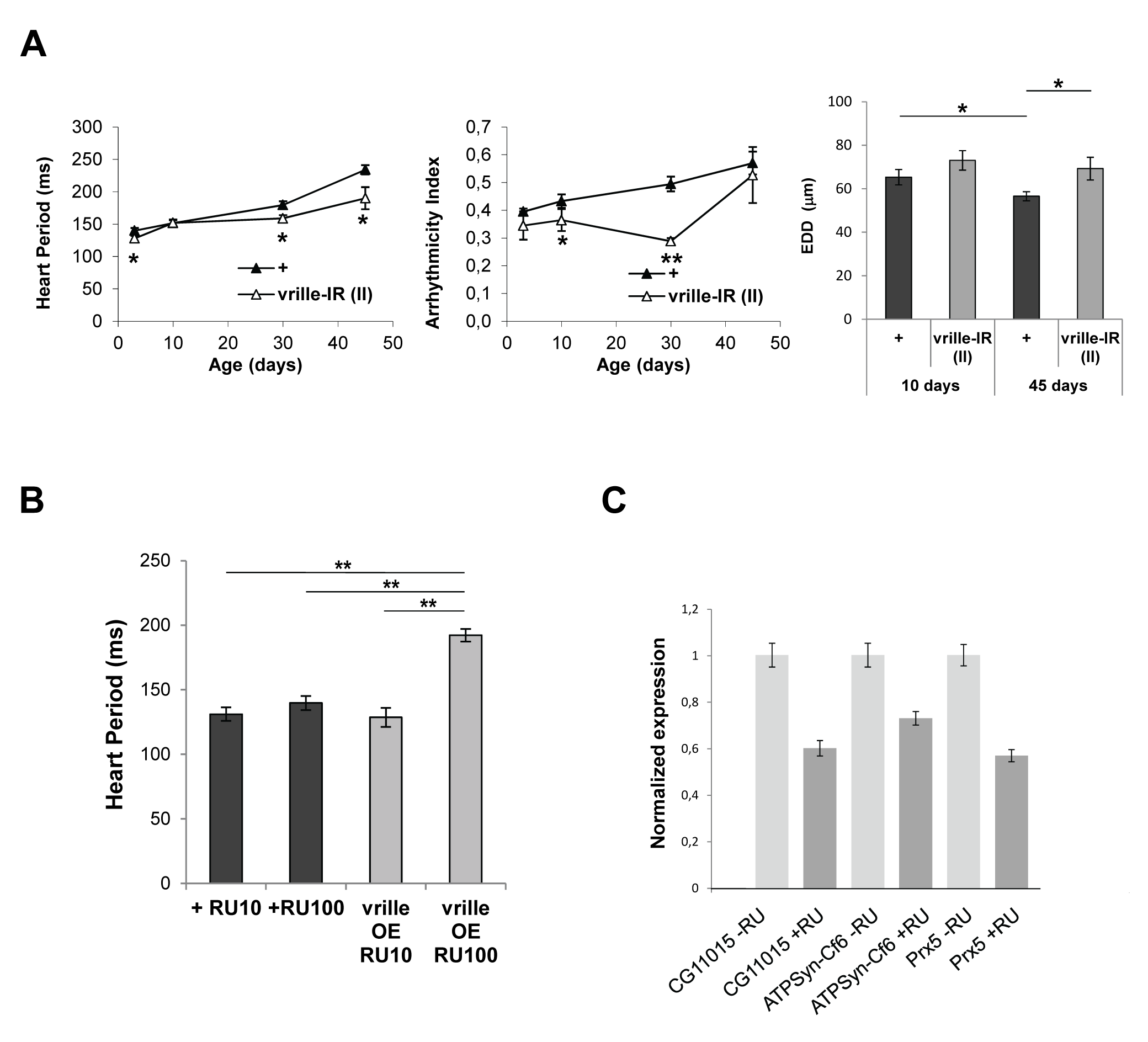

Supplement: Figure S6 — Cardiac Performances in conditions of heart-specific Vri inactivation and overexpression and Validation of putative Vri targets. (A) Heart Period (HP), Arrhytmicity Index (AI) and End Diastolic Diameter (EDD) of w/Y;UAS-mitoGFP/+; Hand-GS/+ (+) and w/Y;UAS-mitoGFP/+;Hand-GS/UAS-vrille-IR(II) (vrille-IR (II)) male flies treated with RU 486 (100 µg/ml of food) during adulthood. All values are means (±SEM). +/3 days: n = 13; +/10 days: n = 26; +/30 days: n = 34; +/45 days: n = 34; vrille-IR (II)/3 days: n = 9; vrille-IR (II)/10 days: n = 11; vrille-IR (II)/30 days: n = 19; vrille-IR (II)/45 days: n = 7. Significant differences with control (+) flies of the same age are indicated * p<5.10−2, ** p<5.10−3. (A) Heart Period (HP) of w/Y;UAS-mitoGFP/+; Hand-GS/+ (+) and w/Y;UAS-mitoGFP/+;Hand-GS/UAS-vrille OE (vrille OE) male flies treated with 10 (RU10) or 100 (RU100) µg of RU486 per ml of food during adulthood. All values are means (±SEM). + RU10: n = 5; + RU100: n = 10; vrille OE RU10: n = 7; vrille OE RU100: n = 10. Significant differences are indicated * p<5.10−2, ** p<5.10−3. (B) 3 genes from cluster 1 (ATPsyn-Cf6 (CG4412), prx5(CG7217) and CG11015, encoding a component of the complex V of the mitochondrial respiratory chain) predicted by cistargetX as potential Vri targets were tested by RQ-PCR following vri overexpression (UAS>Vri) using the inducible driver Da>GeneSwitch. Relative expression between induced (+RU) and non-induced (−RU) was measured. RP49 was used as a reference endogenous gene for normalization. All 3 genes displayed weaker expression following vri overexpression. (TIF) [file pgen.1003081.s006.tif]
