# Supplementary material for: dJun and Vri/dNFIL3 Are Major Regulators of Cardiac Aging in Drosophila
Source: PLoS Genet. 2012 Nov 29;8(11):e1003081. doi: 10.1371/journal.pgen.1003081 (PMC3510041; doi:10.1371/journal.pgen.1003081)
Supplement: Text S1 — Detailed Materials and Methods. (DOCX) [file pgen.1003081.s022.docx]

Supporting Information

**Supporting Materials and Methods**

**Drosophila genetics and molecular biology:**

UAS-Cat (w[1]; P{w[+mC]=UAS-Cat.A}2 ), UAS-Sod1 (w[1]; P{w[+mC]=UAS-Sod.A} B36) [[1](#_ENREF_1)], UAS-dJun-IR (y1 v1; P{TRiP.JF01184}attP2), UAS-dJun OE (y[1]w[1118]; P{w[+mC]=UAS-Jra}2), UAS-vrille-IR (II) (y1 v1; P{TRiP.JF02011}attP2) and UAS-mitoGFP (w[1118]; P{w[+mC]=UAS-mitoGFP.AP}2/CyO) were obtained from the Bloomington Stock Center. UAS-Cat-IR (w[1118]; P{GD1291}v6283), UAS-sgs1-IR (w1118; P{GD10097}v21206), UAS-dJun-IR (II) (w1118 P{GD4400}v10835) and UAS-Kayak-IR (w[1118]; P{GD1469}v6212/TM3)were obtained from the VDRC Stock Center. UAS-Sod1-IR (w; P{w[+mC]=UAS-Sod.IR}3 ) [[2](#_ENREF_2)] and UAS-luciferase were kindly provided by J. Phillips and T. Preat, respectively. UAS-vrille-IR and UAS-vrille[[3](#_ENREF_3)]were kindly provided by R. Terracol. The UAS-CatB transgene was obtained by standard transgenesistechniques after cloning a Xho1-EcoRI fragment of the GH09387 cDNA (gene CG9314) into the pUAST vector. The efficiencies of UAS-dJun-IR and UAS-Cat-IR were tested by RT-q-PCR.

Hand>GeneSwitch transgene: The pP(UAS-GeneSwitch) vector [[4](#_ENREF_4)] was modified to remove the 5XUAS sequence and the Not1 restriction site downstream from the hsp70 promoter, and to introduce single Spe1 and Not1 restriction sites upstream from the hsp70 promoter; the resulting construct contains a short (Mlu1/Spe1/Not1) MCS upstream from the GeneSwitch ORF driven by hsp70 minimal promoter, and was named pHsp70 GeneSwitch. The Hand Delta VM enhancer was excised from PCR2 TOPO (Intron 3 of Hand gene, Delta 1074/1374; from Paululat lab, See [[5](#_ENREF_5)] using Spe1 and Not1 and ligated between the Spe1 /Not 1 in pHsp70 GeneSwitch. Standard P element transformation was used to generate transgenic lines. All seven independent transgenic lines tested demonstrated cardiac-specific expression of GFP when crossed to UAS>GFP lines and induced with RU486. The line displaying the strongest GFP induction was selected for use in this study.

For cardiac imaging experiments, flies were collected within 24 hours of eclosion under brief CO2 anesthesia and housed in groups of 30. Culture conditions and RU treatment were as described in [[6](#_ENREF_6)]. Flies were raised at 26°C under a 12hr-12hr light cycle and transferred every two days onto fresh food. A stock solution (10 mM) of EUK-8 (CALBIOCHEM) was prepared in deionised water and stored in the dark at 4°C. EUK-8 was incorporated in the food medium at a final concentration of 0.2 mM.

**Cardiac Transcriptome Dynamics between 10 days and 40 days:**

- RNA extraction, labeling and microarray hybridization:

Cardiac tubes were hand dissected from aged individuals. For each sample, 10 cardiac tubes were dissected and stored at -80°C in 300 µl of Trizol solution prior to extraction of total RNA. Four samples of 10 cardiac tubes each were generated for each time-point so as to have four biological replicates. RNA extraction, amplification and labeling was performed essentially according to [[7](#_ENREF_7)]. Extracted total RNA (~100 ng) was analyzed using an Agilent 2100 Bioanalyser and RNA 6000 Picochips following the manufacturers’ instructions to monitor RNA quality. mRNA was selectively amplified with the Amino AllylMessageAmp^TM^ II aRNA Amplification Kit (Ambion). The aRNA procedure begins with total RNA that is reverse transcribed using an oligo(dT) primer containing a T7 RNA polymerase promoter sequence. After second strand synthesis, the cDNA serves as template for T7 *in vitro* transcription (IVT). aRNA quality was verified with an Agilent Bioanalyser and subjected to a second round of amplification with a second IVT configured to incorporate the modified nucleotide (amino allyl UTP) into the aRNA for subsequent indirect labeling with fluorescent dyes Cy3 and Cy5. Dye-swap replications, in which each hybridization is done twice, with dye assignments reversed in the second hybridization, were used with a design which allows direct comparison between time points (see scheme). This method allows technical replicates and eliminates variation that might result from differences in fluorescence dye intensities.


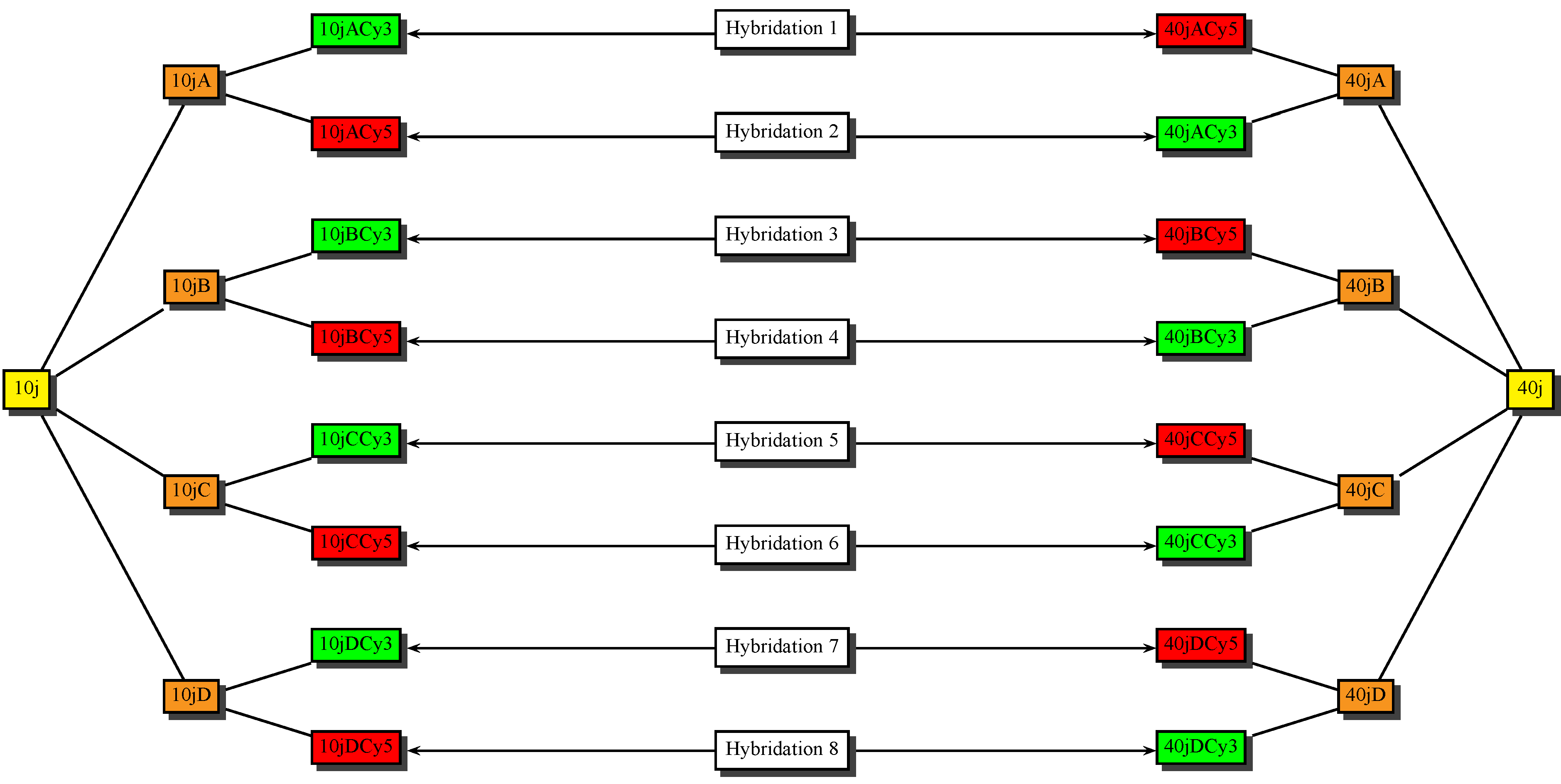


Labeled aRNA samples were mixed in equal proportions and fragmented with the RNA Fragmentation Reagents (Ambion) to enhance aRNA hybridization, and then hybridized on Drosophila Melanogaster HX12 oligonucleotide microarrays (NimbleGen) that contained 137 489 probes (60 mers) representing 16637 genes. After competitive hybridization (using NimBleGen hybridization chambers) at 42°Cfor 16 hours with 150 pmol of dye per sample, the slides were scanned.

- Data treatment and analysis: Lowes normalization of primary expression data was performed. The LIMMA package was used to identify genes with statistically significant differences in gene expression. A predicted false discovery rate of 0.01% was used as the threshold for differential expression. The final dataset comprises 3161 probes representing 1121 unique genes differentially expressed between the two time points.

Gene expression data comparisons were made between our microarray dataset and previously published microarray datasets. Enrichment p-values were based on a test following the hypergeometric distribution. Flymine (<http://www.flymine.org/>) was used to identify statistically relevant over-represented Gene Ontology (GO) and pathway terms in our gene datasets.

- CisTarget X analysis: the cisTargetX method was used essentially as described in [[8](#_ENREF_8),[9](#_ENREF_9)]through the web interface (<http://med.kuleuven.be/cme-mg/lng/cisTargetX/>). The automatic Z score (2.5) was used as the threshold. Clusters 1 and 2 were submitted separately, and over-represented motifs predicted above the threshold are shown in Table S9. Predicted target genes for selected motifs were retrieved using the “candidate targets” link in the result page. **40 days cardiac transcriptome modifications induced by Catalase and dJun genetic manipulation:**
- RNA extraction, labeling and microarray hybridization:

RNA extraction and quantification was performed as above. RNA amplification and labeling was performed using Agilent RNA labeling procedure and hybridized on Agilent 44K Drosophila expression arrays following manufacturer instructions. 3 biological replicates were analyzed for each condition, all being labeled with Cy3 and hybridized on separate arrays.

- Data treatment and analysis: Between arrays quantile normalization of primary expression data was performed. The Rank Product software, included in the TMev package, was used to identify genes with statistically significant differences in gene expression. For Catalase Loss of Function and Gain of Function comparisons, predicted false discovery rate of 0.1% was used as the threshold for differential expression. In the case of dJunRNAi analysis, genes that are downregulated by a factor of at least 1.5 fold were retained for analysis

Gene expression data comparisons were made within our microarray dataset and with previously published microarray datasets. Enrichment p-values were based on a test following the hypergeometric distribution. Flymine (<http://www.flymine.org/>) was used to identify statistically relevant over-represented Gene Ontology (GO) and pathway terms in our gene datasets.

**Q-PCR**:

Confirmation of transcriptome dynamics between 10 and 40 days:200 ng of aRNA was reverse transcribed with 100 U of Reverse transcripase (SuperScript Invitrogen) in a reaction volume of 20 µl. In all cases, three technical replicates were performed on three biological samples for mRNA quantification. The relative abundance of transcripts was assessed using *rp49* to normalize data. Reactions in SYBR®GreenERqPCRSuperMix (Invitrogen) were performed using the CFX® Real-Time PCR Detection System (Bio-Rad Laboratories Inc.). For PCR, 10 ng of cDNA were used together with 200 nM of each primers in 25 µl.

Analysis of the effect of *vri* overexpression on its putative targets: Total RNA was extracted from 10 days old adult flies (w, UAS>Vri; da>GeneSwitch) fed or not with 100 µg/ml RU486 and 1 µg of total RNA was reverse transcribed as above. Q PCR was performed as above.

The sequences of the specific primers are listed in the table below:

| **gene** | **forward primer** | **reverse primer** |
| --- | --- | --- |
| Catalase | acaagatgctgcatggtcgtc | tcctggttgtccgtcacattc |
| ATPsyn-Cf6 | tccagttccccgatgttaag | cgaatcgcttgttgttttcc |
| Prx5 | acgatccctttgtgatgtcc | gacattcagctcggtcacct |
| CG11015 | attggttcaagctggtggag | gccctttcactcctctctgtt |
| CG6981 | agggttacatgtccgacgag | tgcgattggtatccccacta |
| rost | gtgacctttgtgggcatctt | ggtgcccatgaggtgatatt |
| tsp42Ed | tggagaagatcgtccagacc | gttagtgcgcaggttccatt |
| dJun | agactgaaaccccctcgaat | gctgttcgttatcgctctcc |
| rp49 | gacgcttcaagggacagtatctg | aaacgcggttctgcatgag |

***In vivo* imaging of fly hearts.**

Flies, expressing the GFP protein targeted to mitochondria (mitoGFP) to label the cardiac tube,were briefly anesthetized with Triethylamine (Flynap, Carolina Biological Supply Co) as described in [[10](#_ENREF_10)], fixed by their wings onto glass slides (Figure S2, A) and observed with a Zeiss SteREO Lumar.V12 Stereomicroscope, using a NeoLumar S 1.5× objective. Video movies were acquired with an AxioCamHR Camera (32 frames per second, 1000 frames per movie, frame size 173,5 x 694 μm).

**Automated detection and quantification of heartbeat**

For every video, the 1000 frames were flattened into one by using the ImageJ function Zproject ([Max Intensity]). The picture generated wasthresholded for light intensity by using the set AutoThreshold function. The anterior part of the heart (abdominal segments A1/A2) was then detectedwith the Analyze Particles tool from ImageJ (minimum size = 200, maximum size = 9000, circularity = 0 – 0.99). The automatic positioning of the acquisition zone used the XM variable as the abscissa origin for the function MakeRectangle to generate a 1 pixel wide and 50 pixel high (the height the video field) row. M-Modes were generated by horizontal alignment of rows extracted at the same position from each movie frame (see Figure S2, B and C). A flattened picture showing the acquisition row was generated for each video for subsequent verification. The row was manually repositioned when required to improve the M-Mode quality (in this study for less than 8% of the movies). The macro was developed and tested using ImageJ 1.43s (NIH, USA).

An image processing algorithm was developed to generate cardiograms from M-mode images with Matlab R2010b (The Mathworks). Cardiograms are defined by the distance between the two maxima of mitochondrial fluorescence at each timepoint. The first step of the algorithm finds the position of the heart in M-mode images for each time point (red line in Figure S2 D). These positions are obtained using filtering and mathematical morphology [[11](#_ENREF_11)]as follows. Median filter is used to eliminate noise from the spatial dimension for each time point. A threshold using the Otsu’s method combined with mathematical morphology operators for filling holes is applied to a sliding window of 30 pixels width along the temporal axis to obtain binary images. This mask is used to identify the median position of the heart. Local maxima are then localized on each side of the median position of the heart (green plots in Figure S2 D). In the rare cases where one of the two maxima cannot be determined by the algorithm, the missing position is assumed to be at the same distance from the median position of the heart as the observed position on the other side. The cardiogram is finally generated by calculating distances between the two maxima for each time point. The second step of the algorithm computes the temporal position for each end-systolic and end-diastolic position of the heart by finding all local maxima and minima on the cardiogram. The resulting file from the previous analysis was incorporated in an Access DataBase to extract, for each cardiogram, higher order parameters. During this process, to take into account the limited spatial resolution of our system and prevent the detection of spurious events, we filtered the data so that the differences in amplitudes between consecutive diastolic and systolic positions are larger than 2 pixels. We then extracted the mean and standard deviation values for the heart period (HP, calculated as the duration between consecutive end-diastolic positions) and the End Diastolic diameter. An Arrhythmicity Index (AI) was calculated as the standard deviation of the HP normalized to the median HP [[12](#_ENREF_12)]. For each experimental condition, the distribution and the mean and standard deviation values of the HP and AI obtained from the relevant movies were calculated by appropriate requests. Statistical significance was assessed by non-parametric Wilcoxon analysis.The statistical significance between the slope of curves for HP or AI obtained in two different condition was assessed by the method of Zar[[13](#_ENREF_13)]), implemented in the GraphPad Prism package.

**References:**

1. Anderson PR, Kirby K, Hilliker AJ, Phillips JP (2005) RNAi-mediated suppression of the mitochondrial iron chaperone, frataxin, in Drosophila. Hum Mol Genet 14: 3397-3405.

2. Missirlis F, Hu J, Kirby K, Hilliker AJ, Rouault TA, et al. (2003) Compartment-specific protection of iron-sulfur proteins by superoxide dismutase. J Biol Chem 278: 47365-47369.

3. Szuplewski S, Kottler B, Terracol R (2003) The Drosophila bZIP transcription factor Vrille is involved in hair and cell growth. Development 130: 3651-3662.

4. Osterwalder T, Yoon KS, White BH, Keshishian H (2001) A conditional tissue-specific transgene expression system using inducible GAL4. Proc Natl Acad Sci U S A 98: 12596-12601.

5. Popichenko D, Sellin J, Bartkuhn M, Paululat A (2007) Hand is a direct target of the forkhead transcription factor Biniou during Drosophila visceral mesoderm differentiation. BMC Dev Biol 7: 49.

6. Tricoire H, Battisti V, Trannoy S, Lasbleiz C, Pret AM, et al. (2009) The steroid hormone receptor EcR finely modulates Drosophila lifespan during adulthood in a sex-specific manner. Mech Ageing Dev 130: 547-552.

7. Zeitouni B, Senatore S, Severac D, Aknin C, Semeriva M, et al. (2007) Signalling pathways involved in adult heart formation revealed by gene expression profiling in Drosophila. PLoS Genet 3: 1907-1921.

8. Aerts S, Quan XJ, Claeys A, Naval Sanchez M, Tate P, et al. (2010) Robust target gene discovery through transcriptome perturbations and genome-wide enhancer predictions in Drosophila uncovers a regulatory basis for sensory specification. PLoS Biol 8: e1000435.

9. Potier D, Atak ZK, Sanchez MN, Herrmann C, Aerts S (2012) Using cisTargetX to Predict Transcriptional Targets and Networks in Drosophila. Methods Mol Biol 786: 291-314.

10. Paternostro G, Vignola C, Bartsch DU, Omens JH, McCulloch AD, et al. (2001) Age-associated cardiac dysfunction in Drosophila melanogaster. Circ Res 88: 1053-1058.

11. Serra J, editor (1982) Image Analysis and Mathematical Morphology: Academic Press.

12. Fink M, Callol-Massot C, Chu A, Ruiz-Lozano P, Izpisua Belmonte JC, et al. (2009) A new method for detection and quantification of heartbeat parameters in Drosophila, zebrafish, and embryonic mouse hearts. Biotechniques 46: 101-113.

13. Zar J (1984). Biostatistical Analysis. 2nd ed: Prentice-Hall.
